# Co-designing meaningful extended reality for physical rehabilitation: a stakeholder-driven approach to embodied telehealth

**DOI:** 10.3389/frvir.2025.1675309

**Published:** 2026-01-27

**Authors:** Aviv Elor, Adrian Parrales, Strauss Michael Bourdon, Maxim Kuznetsov, Kamryn Callwood, Alyssa Tu, Michael Powell, Ash Robbins, Matthew Bundle, Felicia Skelton, Hilary Touchett

**Affiliations:** 1 Immergo Labs, Inc., Mountain View, CA, United States; 2 Department of Medicine, Baylor College of Medicine, Houston, TX, United States; 3 School of Integrative Physiology and Athletic Training, University of Montana, Missoula, MT, United States; 4 Spinal Cord Injury Care Line, Michael E. DeBakey VA Medical Center, Houston, TX, United States; 5 Ben Taub Department of Physical Medicine and Rehabilitation, Baylor College of Medicine, Houston, TX, United States; 6 Houston VA HSR&D Center for Innovations in Quality, Effectiveness and Safety (IQuESt), Michael E. DeBakey VA Medical Center, Houston, TX, United States; 7 South Central Mental Illness Research, Education and Clinical Center, a Virtual Center, Houston, TX, United States

**Keywords:** accessibility, co-design, embodied telehealth, extended reality, immersive virtual reality, physical therapy, physical rehabilitation, user-centered design

## Abstract

**Introduction:**

Extended Reality (XR) technologies offer unprecedented opportunities to redefine physical rehabilitation experiences through embodied telepresence and full-body motion tracking. Realizing meaningful XR in physical rehabilitation requires stakeholder input addressing remote embodied therapy’s unique design challenges.

**Methods:**

This study presents one of the first stakeholder co-design workshops on embodied telehealth for physical rehabilitation, engaging participants [N = 24 total including ten clinicians, eight patients, three designers, and three engineers] across five focus groups with stakeholders from clinical, academic, and industry settings. Through structured usability testing and a four-axis framework analysis, we evaluated immersive Virtual Reality (VR) applications supporting synchronous clinician-led embodied appointments and asynchronous clinician-authored patient home exercise programs with full-body tracked avatars and biomechanical assessments.

**Results:**

Stakeholder feedback reinforced the need for embodied agency with adaptive input modalities for diverse patient needs, ensuring clinical authenticity through real-time full-body tracking for accurate movement correction and scaling interaction complexity from minimal interfaces for VR novices (78% of participants) to customizable clinical dashboards. Crossstakeholder prioritization identified minimalist XR interface navigation (19 votes) and flexible clinical assessment capabilities (17 votes) as the highest-priority design requirements, while exit surveys suggested high patient comfort (M = 4.50/5) and strong clinician adoption interest (M = 4.14/5).

**Discussion:**

We propose six design principles for meaningful XR telehealth in physical rehabilitation: 1) embodied guidance with real-time feedback, 2) progressive complexity with minimalist defaults, 3) adaptive accessibility through multi-modal input, 4) clinical authenticity via domain-specific assessments, 5) biomechanical precision for trust and safety, and 6) contextual onboarding to improve therapeutic competency. These findings offer design considerations for developing embodied XR telehealth systems that support sustained therapeutic engagement and meaningful rehabilitation outcomes.

## 1 Introduction

Extended Reality (XR) technologies, particularly immersive Virtual Reality (VR), can redefine how physical rehabilitation is delivered by enabling embodied telepresence, full-body motion tracking, and AI-driven biomechanical assessment. These advancements can potentially improve access to specialized care, enhance patient engagement, and provide clinicians with quantifiable movement data to guide remote interventions (Riva et al., 2019; Matamala-Gomez et al., 2021; Elor and Kurniawan, 2020b). However, despite rapid technological progress, XR-based rehabilitation platforms face significant adoption barriers, including usability challenges, accessibility concerns, and workflow integration gaps within clinical settings (Greenhalgh et al., 2017; Prats-Bisbe et al., 2024).

### 1.1 Research context and motivation

Considering these challenges, this paper aims to contribute to the emerging field of Meaningful XR, experiences that create lasting cognitive, emotional, or behavioral changes beyond the moment of use for physical rehabilitation. This work explores a co-design approach to prioritize user-centered systems by ensuring that stakeholder needs and values directly shape the XR experience through participatory design rather than pure technology capabilities driving user requirements (Antoniou et al., 2024).

Existing XR rehabilitation tools are often developed without direct input from end-users, potentially leading to interfaces that may be difficult to navigate, unintuitive interactions, and features that lack clinical relevance or importance (Cucinella et al., 2025; Mehrabi et al., 2021). While VR and computer vision motion tracking offer new ways to deliver remote rehabilitation, their success depends on how well these systems align with patients’ and clinicians’ cognitive, physical, and clinical needs (Eisapour et al., 2018). Co-design methodologies, which involve end-users in the design process, have been recognized as a crucial approach for ensuring that emerging XR technologies are not only innovative but can also be practical, user-friendly, and clinically effective (Ng et al., 2024; Shah et al., 2023).

### 1.2 Study approach and objectives

This study presents the findings from a cross-industry and academic co-design workshop conducted in August 2024 involving Immergo Labs, Baylor College of Medicine, the University of Montana, and United States physical rehabilitation community members. The workshop aimed to evaluate and refine the usability, accessibility, and clinical applicability of an VR and web-based physical rehabilitation platform prototype that supports embodied telehealth appointments between physical rehabilitation clinicians and patients using full-body avatars, AI-driven biomechanical assessment, and clinician-authored asynchronous exercise programs with patient movement guidance. Specifically, this study explored the following aims:

1. Evaluate User Experience and Interaction Design: Assess the usability of prototype VR and web applications for real-time clinician-patient interactions and asynchronous exercise tracking through streamlined usability assessments suitable for early-stage co-design contexts.
2. Identify Barriers to Adoption: Understand clinician and patient pain points related to input methods, user onboarding, and workflow integration through structured co-design methodologies (Antoniou et al., 2024).
3. Prioritize Future Design Enhancements: Use structured feedback frameworks to prioritize critical improvements in UI/UX, motion tracking, and clinical decision support tools by stakeholder importance.
4. Establish User-Centered Design Principles for Embodied XR Telehealth: Contribute insights for improving immersive telehealth platforms that leverage AI and VR technologies for meaningful user experience (Riva et al., 2019).

Through direct collaboration with physical therapists, rehabilitation clinicians, patients with lived experiences, product designers, and product engineers, this study informs the design and development of immersive clinically relevant embodied telehealth platforms. The findings aim to establish foundations for meaningful XR design principles in healthcare contexts, demonstrating how co-design processes can identify critical requirements for creating empowering therapeutic technologies that extend beyond momentary engagement to create lasting health improvements and behavioral change. The broader impact lies in providing user-centered design recommendations for developing accessible and scalable immersive telehealth applications, informing considerations for the next-generation of AI-enhanced XR rehabilitation platforms.

## 2 Related works

XR encompasses immersive technologies that blend physical and virtual environments, including VR, Augmented Reality (AR), and Mixed Reality (MR). In recent years, XR advancements have spurred research into its potential applications in healthcare, particularly in physical rehabilitation (Keshner et al., 2019; Castillo et al., 2024). Traditional rehabilitation methods, while effective, often suffer from patient compliance challenges (Campbell et al., 2001; O’Carroll and Hendriks, 1989; Sluijs et al., 1993). XR technologies offer innovative ways to improve engagement, adherence, and treatment outcomes by providing interactive and data-driven rehabilitation experiences that can create meaningful, lasting changes in patient behavior and motor function (Riva et al., 2019).

### 2.1 Meaningful XR experiences in rehabilitation contexts

Meaningful XR extends beyond traditional usability metrics to encompass experiences that create lasting physical health improvements and behavioral changes (Riva et al., 2019). Riva et al. (2019) establish the theoretical framework for meaningful VR through embodied cognition principles, proposing that VR’s effectiveness stems from shared mechanisms with brain processes, or embodied simulations, making it uniquely capable of creating meaningful experiences that can alter bodily processes and enhance wellbeing. This work introduces “Embodied Medicine” as a paradigm for designing virtual environments that modify experiences from both external and internal perspectives, providing essential theoretical grounding for meaningful XR applications in rehabilitation. In this study, we draw on this framework to guide stakeholder co-design activities, using participant feedback to identify design requirements that stakeholders perceive as essential for creating rehabilitation experiences extending beyond momentary engagement.

Recent implementations demonstrate the practical realization of meaningful XR in healthcare contexts. Maltby et al. (2023) deployed VR training modules across 23 rural hospitals for stroke telehealth, achieving high acceptability scores and demonstrating VR’s capacity to create meaningful remote healthcare experiences. Similarly, Jonsdottir et al. (2021) validated the continuity of meaningful VR rehabilitation from clinical to home environments, achieving 89% adherence rates and significant functional improvements, establishing frameworks for meaningful, long-term VR rehabilitation that bridges institutional and home settings. These advances build upon foundational work in therapy game design, where structured approaches to creating engaging, therapeutically meaningful interactions have been systematically developed (Duval, 2020; Duval et al., 2022).

### 2.2 Telerehabilitation and its limitations

Telerehabilitation, often referred to as TeleRehab or rehabilitation oriented telehealth, leverages digital platforms to extend access to rehabilitation services (Niknejad et al., 2021). In this model, physical therapists interact with patients through synchronous videoconferencing or asynchronous video-based guidance (Niknejad et al., 2021). The adoption of TeleRehab surged during the COVID-19 pandemic, demonstrating its feasibility while also revealing critical limitations (Elor et al., 2022; Matamala-Gomez et al., 2021). One major drawback was the lack of physical interaction, which hindered clinicians’ ability to assess range of motion, strength, and injury severity. Many therapists reported feeling more like “life coaches” than healthcare professionals, as the inability to physically manipulate a patient’s movement or observe biomechanics comprehensively limited their capacity to diagnose and treat patients effectively (Elor et al., 2022). Beyond clinical interaction challenges, telerehabilitation systems face infrastructural constraints including network instability, bandwidth limitations, and video latency that can disrupt real-time therapeutic guidance (Arlati et al., 2025; Antoniou et al., 2022). These challenges underscore the need for immersive technologies to solve the gap between in-person and remote rehabilitation through embodied telepresence that maintains meaningful therapeutic interactions (Elor et al., 2022; Matamala-Gomez et al., 2021).

### 2.3 Effectiveness of virtual reality in physical rehabilitation

Research on VR-based physical rehabilitation has demonstrated promising results across various conditions, including stroke recovery (Chen et al., 2022; Demeco et al., 2023; Peláez-Vél et al., 2023), Parkinson’s disease (Feng et al., 2019; Gulcan et al., 2023; Pazzaglia et al., 2020; Sarasso et al., 2022), and chronic lower back pain (Afzal et al., 2022). Recent meta-reviews confirm VR’s effectiveness in creating meaningful rehabilitation experiences that extend beyond traditional therapy approaches (Keshner et al., 2019).

Compared to traditional rehabilitation, immersive VR therapy offers:

- Enhanced patient engagement: Gamified environments with enhanced user presence can increase motivation and adherence while creating meaningful experiences (Shah et al., 2023; Muñoz et al., 2022).
- Objective motion tracking: AI-assisted tracking provides quantitative movement analysis for meaningful progress assessment (Castillo et al., 2024).
- Real-time feedback: Immediate corrections improve motor learning and rehabilitation outcomes through embodied interaction (Riva et al., 2019).
- Scalability: Remote accessibility reduces geographical and logistical barriers while maintaining meaningful therapeutic relationships (Jonsdottir et al., 2021).

Recent studies have further examined factors influencing patient adherence and long-term engagement in VR rehabilitation, demonstrating that usability, acceptability, and motivational design significantly impact sustained participation across diverse populations including elderly patients with chronic conditions and post-COVID recovery (Colombo et al., 2019; Mondellini et al., 2023). Despite these advantages, successful VR implementation in rehabilitation requires careful attention to user interface (UI) design, input methods, and accessibility features. The effectiveness of VR rehabilitation tools is heavily dependent on both hardware (e.g., tracking accuracy, controller usability) and software (e.g., workflow integration, UI intuitiveness), highlighting the importance of user-centered design in XR development (Sutcliffe and Gault, 2004; Kim and Rhiu, 2024).

### 2.4 Co-design methodologies in XR rehabilitation

A growing body of research advocates for co-design methodologies to ensure that XR rehabilitation platforms align with the needs of the clinician and patient while creating meaningful experiences (Brassel et al., 2021; Antoniou et al., 2024). Co-design involves direct collaboration with end users, including patients, clinicians, and technology developers, to iteratively refine a product. A systematic review on VR rehabilitation for acquired brain injuries emphasized the need for user-centered design and interdisciplinary collaboration to maximize effectiveness (Brassel et al., 2021).

Recent co-design studies demonstrate the critical importance of multi-stakeholder engagement. Muñoz et al. (2022) conducted a comprehensive co-design involving seven persons living with dementia/MCI, five exercise professionals, five community-dwelling older adults, and multidisciplinary research teams, showing how different stakeholder types contribute unique perspectives through iterative design workshops. Their collaborative approach resulted in VR games that showed similar efficacy to human-guided exercises while maintaining high user engagement. Duval et al. (2022) further demonstrate this principle through clinician-centered design of gesture-based VR games for stroke rehabilitation, emphasizing the importance of involving healthcare professionals directly in game design decisions to ensure therapeutic validity.

Shah et al. (2023) demonstrated multifaceted co-design processes involving elderly participants and therapists, revealing that collaboration between users proved more effective than competitive elements in VR rehabilitation. Their stakeholder-driven design decisions throughout development achieved usability scores of 83.75 ± 13.3 and high user satisfaction, establishing evidence for social collaboration aspects in meaningful VR rehabilitation. Such approaches may align with broader participatory design approaches in health technology that emphasize research-through-design methodologies for wellness applications (Duval, 2022).

Despite this growing evidence, there is are limited standardized co-design frameworks for XR-based rehabilitation tools. Previous studies have attempted various approaches with differing levels of stakeholder involvement:

- Limited stakeholder input: Some VR co-design studies involved only health professionals and engineers, omitting patient perspectives (Bryant et al., 2024).
- Minimal involvement of the patient: In a co-designed VR game for patients with cognitive dysfunction from major depressive disorder, patients and clinicians participated only in a 1-h teleconference and did not actively participate in the software (Hernandez Hernandez et al., 2023).
- Comprehensive co-design approaches: Other studies, such as VR applications for autism spectrum disorder, traumatic brain injury, and palliative care, incorporated stakeholder feedback at both pre-design and post-design stages (Gabrielli et al., 2023; Nunnerley et al., 2023; Ng et al., 2024), demonstrating more iterative and effective co-design models.

### 2.5 Stakeholder engagement and participatory design frameworks

Recent research emphasizes the critical importance of authentic stakeholder engagement in healthcare XR development. Antoniou et al. (2024) identify five critical themes for effective co-creation: authentic stakeholder engagement, organizational rigor, authentic communication through face-to-face collaboration, balanced participant composition, and educational rigor. Their research emphasizes participatory design methods as crucial for reducing development time and enabling the smooth transfer of educator requirements to technical specifications.

Ng et al. (2024) demonstrate a participatory design involving six clinical psychologists with practical life review therapy experience, emphasizing the critical importance of involving healthcare professionals as end-users. Their research applies key participatory design principles, including collaborative work, critical reflection, enrichment of all collaborators, and equal importance of process and outcome, providing methodological frameworks for ethical healthcare technology development.

Cucinella et al. (2025) employed comprehensive human-centered design approaches, including observational studies, interviews with 11 neurorehabilitation experts, online questionnaires with 24 experts, and participatory design workshops with eight experts to co-create VR training environments. Their approach emphasizes understanding existing rehabilitation practices before designing virtual equivalents, demonstrating the importance of domain expertise integration in healthcare XR design.

### 2.6 Evaluation approaches for early-stage XR design

While comprehensive usability frameworks like User Satisfaction Evaluation Questionnaire (USEQ) (Gil-Gómez et al., 2017) and Virtual Reality System Usability Questionnaire (VRSUQ) (Kim and Rhiu, 2024) provide detailed assessment capabilities for mature VR rehabilitation systems, early-stage co-design processes require streamlined evaluation approaches that prioritize stakeholder engagement over extensive measurement (Eisapour et al., 2018). Structured feedback frameworks, such as a four-axis feedback framework (exploring design areas to Raise, Reduce, Eliminate, Create), can enable systematic collection of design insights while maintaining collaborative focus during participatory design workshops (Antoniou et al., 2024). This approach aligns with participatory design principles where understanding user needs and design opportunities can often take precedence over detailed usability metrics during initial prototype development phases (Duval, 2022).

### 2.7 A brief background on immergo

Immergo Labs^1^, a startup funded by the United States National Science Foundation (NSF) Small Business Innovation Research (SBIR) program (Lanahan, 2016), is developing a novel immersive VR platform for telehealth-based physical rehabilitation inspired by nearly a decade of research on XR rehabilitation (Elor et al., 2018a; Elor et al., 2018b; Elor et al., 2019; Elor et al., 2020; Elor and Kurniawan, 2020a; Powell et al., 2020; Elor and Kurniawan, 2020b; Elor et al., 2021; Elor and Ward, 2021; Elor, 2021; Elor et al., 2022; Duval et al., 2022; Powell et al., 2022; Kurniawan et al., 2024). This study builds upon previous research by adopting a structured co-design methodology that actively engages patients, clinicians, product designers, and engineers to refine an embodied telehealth solution that emphasizes real-world usability testing, iterative feedback loops, and interdisciplinary collaboration to ensure that the final product meets clinical and technological requirements while creating meaningful user experiences. As highlighted in previous studies, 2D telehealth displays often struggle from a lack of objective measurements and challenging remote interactions (Elor et al., 2022; Matamala-Gomez et al., 2021). Immergo is iteratively developing an XR platform to address these challenges, with co-design participants engaging with platform prototypes in this study’s usability tasks.

The prototypes evaluated in this study were developed iteratively over approximately 3 months during Immergo’s Phase I NSF SBIR, informed by biweekly playtesting sessions with a panel of 10 physical therapists who provided informal feedback guiding feature development as well as over 100 interviews with physical therapists (Elor et al., 2022). The co-design workshop reported in this article was conducted at the start of Immergo’s Phase II NSF SBIR to gather structured input from diverse stakeholders, including patients, before finalizing development for in-the-wild deployment. This approach aligns with user-centered design principles emphasizing iterative refinement across multiple development phases rather than single-point user involvement (Norman, 2013).

### 2.8 Contributions of this study

Specifically, this study expands upon the discussed related works by:

1. Implementing a comprehensive co-design process towards embodied telehealth: Actively engaging a diverse group of stakeholders (clinicians, patients, engineers, and designers) in structured usability testing, prioritization exercises, and iterative refinement using established participatory design methodologies (Antoniou et al., 2024).
2. Addressing key barriers in meaningful XR rehabilitation: Providing insights into UI/UX challenges, input methods, and full-body tracking improvements that support embodied telepresence (Riva et al., 2019).
3. Bridging the gap between research and industry: Collaborating across academic (Baylor College of Medicine, University of Montana) and industry (independent clinician practitioners and Immergo Labs) partners to develop a scalable, evidence-based XR rehabilitation platform that creates meaningful user experiences.
4. Demonstrating evaluation methodologies for early-stage XR healthcare application co-creation: Using streamlined four-axis framework analysis (Kim, 2005) and focused usability assessment to collect and organize stakeholder feedback systematically while maintaining collaborative workshop engagement.

The findings from this study provide design recommendations for future XR rehabilitation tools and contribute to the broader user-centered embodied telehealth design considerations for digital health with emerging technologies.

## 3 Methods

### 3.1 Approach and rationale

This study employs co-design as its primary methodological lens, understood here as a subset of participatory design that emphasizes direct collaboration between end-users and designers in shaping product features and interactions (Sanders and Stappers, 2008). Our evaluation approach combines participatory design principles with streamlined usability assessment optimized for early-stage co-design contexts. Rather than comprehensive usability instruments that can overwhelm prototype evaluation sessions, we employ focused single ease-of-use ratings and structured four-axis framework analysis to maintain collaborative engagement while systematically capturing stakeholder feedback (Kim, 2005; Antoniou et al., 2024). This approach prioritizes understanding user needs and design opportunities over detailed usability metrics, consistent with early-stage participatory design best practices where stakeholder engagement depth takes precedence over measurement precision (Duval, 2022; Eisapour et al., 2018).

### 3.2 Stakeholder workshop design

**FIGURE 1:**
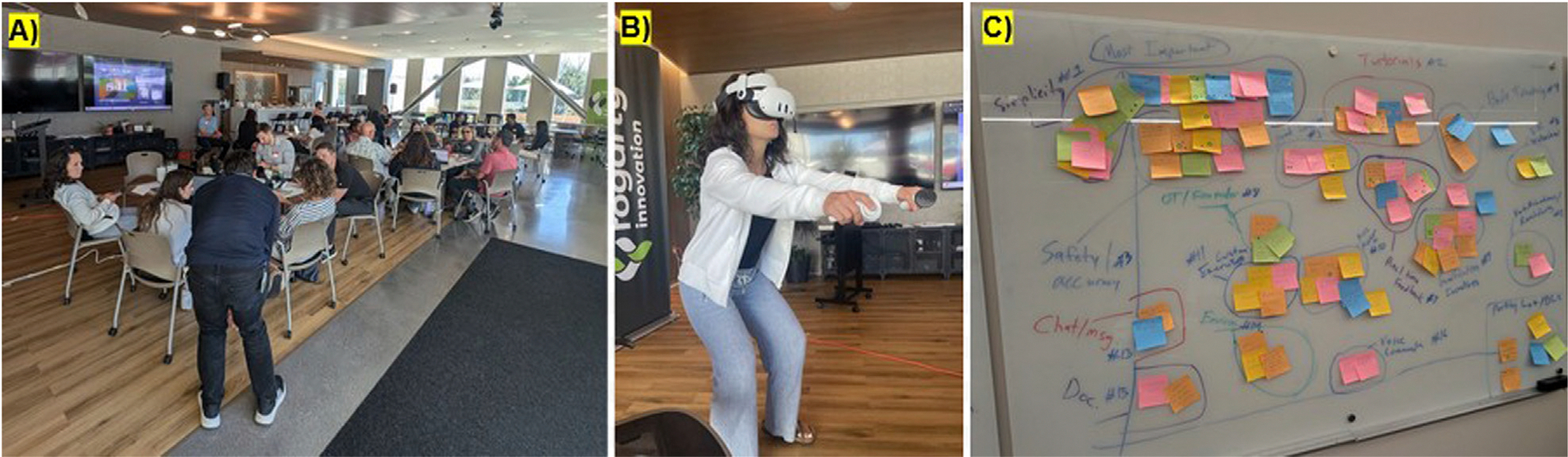
Stakeholder workshop setting at Fogarty Innovation including **(A)** Layout 5 focus group teams (3 clinicians and 2 patients) for the full-day workshop. **(B)** A stakeholder trying Immergo’s VR exercise program prototype. **(C)** Focus group design feedback sticky notes grouped by themes from most important (top) to least important (bottom) with voting dots from other teams used to inform the Immergo product team.

Research participants engaged in a full-day stakeholder co-design workshop on 26 August 2024, at Fogarty Innovation^2^ in Mountain View, California, from 9:00 a.m. to 5:00 p.m. PST. The primary objective was to evaluate and refine the usability, accessibility, and clinical relevance of the Immergo VR and web-based rehabilitation platform through meaningful stakeholder engagement. The session engaged participants in structured usability testing, design thinking exercises, and collaborative prioritization activities to balance clinical validity with user experience considerations (Duval et al., 2022). Figure 1 shows the workshop environment and a sticky note thematic clustering generated during the workshop.

### 3.3 Participants

A total of 24 stakeholders participated in the workshop, divided into five focus groups following established multi-stakeholder co-design principles (Muñoz et al., 2022). Each group included 3–5 members with a total of three clinician-focused groups and two patient-focused groups to ensure balanced representation. Participant demographics and experience distribution are summarized in Table 1 and below.

- 10 Clinicians: Physical therapists, occupational therapists, and clinician researchers with diverse specialization areas, including orthopedic (67%), geriatric (67%), neurological (44%), and sports rehabilitation (44%).
- 8 Patients: Individuals with prior lived experiences in physical rehabilitation, primarily engaged in injury recovery (78%) and surgical recovery (33%).
- 6 Industry Representatives: three user researchers/designers and three engineers from Immergo Labs, serving dual roles as domain experts and workshop facilitators.
- 5 Table Moderators: Immergo product team members who facilitated technology setup, supported group discussions, and guided usability tasks while maintaining neutrality in design feedback collection.
- 1 Room Moderator: Overall workshop coordinator responsible for agenda management, timing, and cross-group troubleshooting support.

Each team had at least one dedicated Immergo product team member serving as a moderator, responsible for timekeeping, supporting usability exercises, taking detailed notes, and casting VR headset views to laptops for group observation during individual VR interactions. This setup enabled collective observation and discussion of user experiences, facilitating shared understanding across diverse stakeholder perspectives (Shah et al., 2023).

The demographic distribution of the participants was designed to reflect the target user population for possible embodied XR telehealth platforms. The clinician-to-patient ratio (10:8) provided balanced representation of the stakeholders, ensuring that the perspectives of both the provider and the end-user were adequately captured without either group dominating the design discussion (Antoniou et al., 2024). The diversity of specialization of the clinician, with a strong representation in orthopedic rehabilitation (67%) and geriatric (67%) along with neurological (44%) and sports medicine (44%), reflects the breadth of physical rehabilitation practice areas that can benefit from integrated telehealth solutions. The age distribution, centered primarily in the 35–44 range (39%) with representation across young adults to older adults, captures the working-age clinician population and diverse patient demographics typical of physical rehabilitation settings. The gender distribution (42% male, 59% female) approximates physical therapy workforce demographics, where approximately two-thirds of physical therapists are women (American Physical Therapy Association, 2023).

The distribution of VR experience, with 61% of participants having limited or no prior VR exposure (39% none, 22% limited), represents the expected early adopter population for clinical XR systems, since most rehabilitation clinicians and patients currently have minimal VR experience (Elor et al., 2022). Capturing qualitative user perceptions and usability barriers from VR novices provides insights for designing accessible onboarding experiences that do not assume prior technical expertise. The high video call experience (89% experienced or highly experienced) reflects widespread adoption of telehealth during and after the COVID-19 pandemic (Matamala-Gomez et al., 2021). This provided participants with a baseline understanding of remote healthcare delivery that informed their evaluation of embodied telepresence.

### 3.4 Technology setup and materials

**FIGURE 2:**
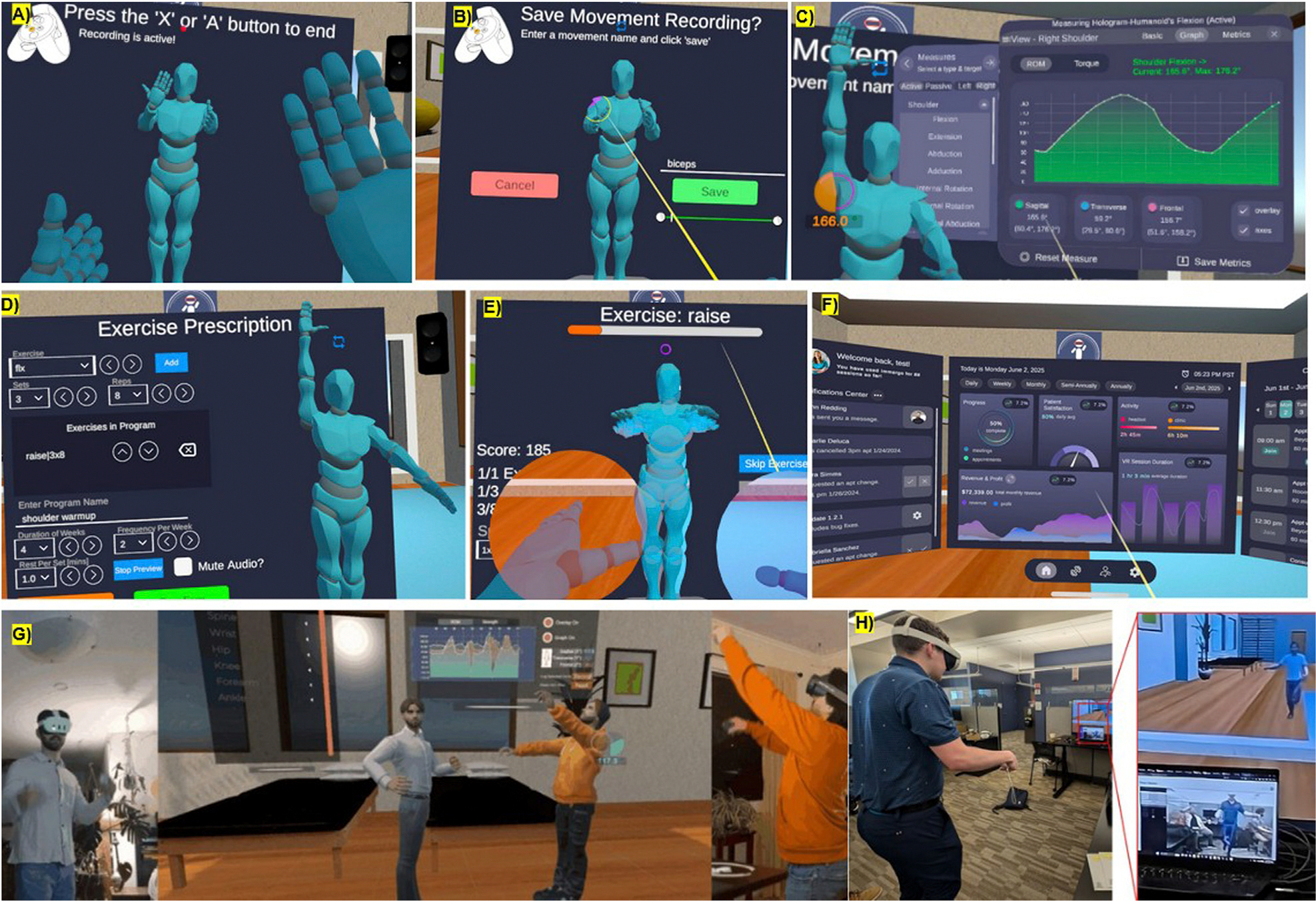
VR Application Prototype Usability Tasks including **(A)** mirrored movement recordings, **(B)** movement recording preview and crop save tool, **(C)** real-time measurement movement evaluation tool, **(D)** exercise program creation, **(E)** exercise program player, **(F)** progress dashboard, **(G)** two users meeting in the immergo app, and **(H)** a user using full body tracking in VR with Immergo’s webapp.

**FIGURE 3:**
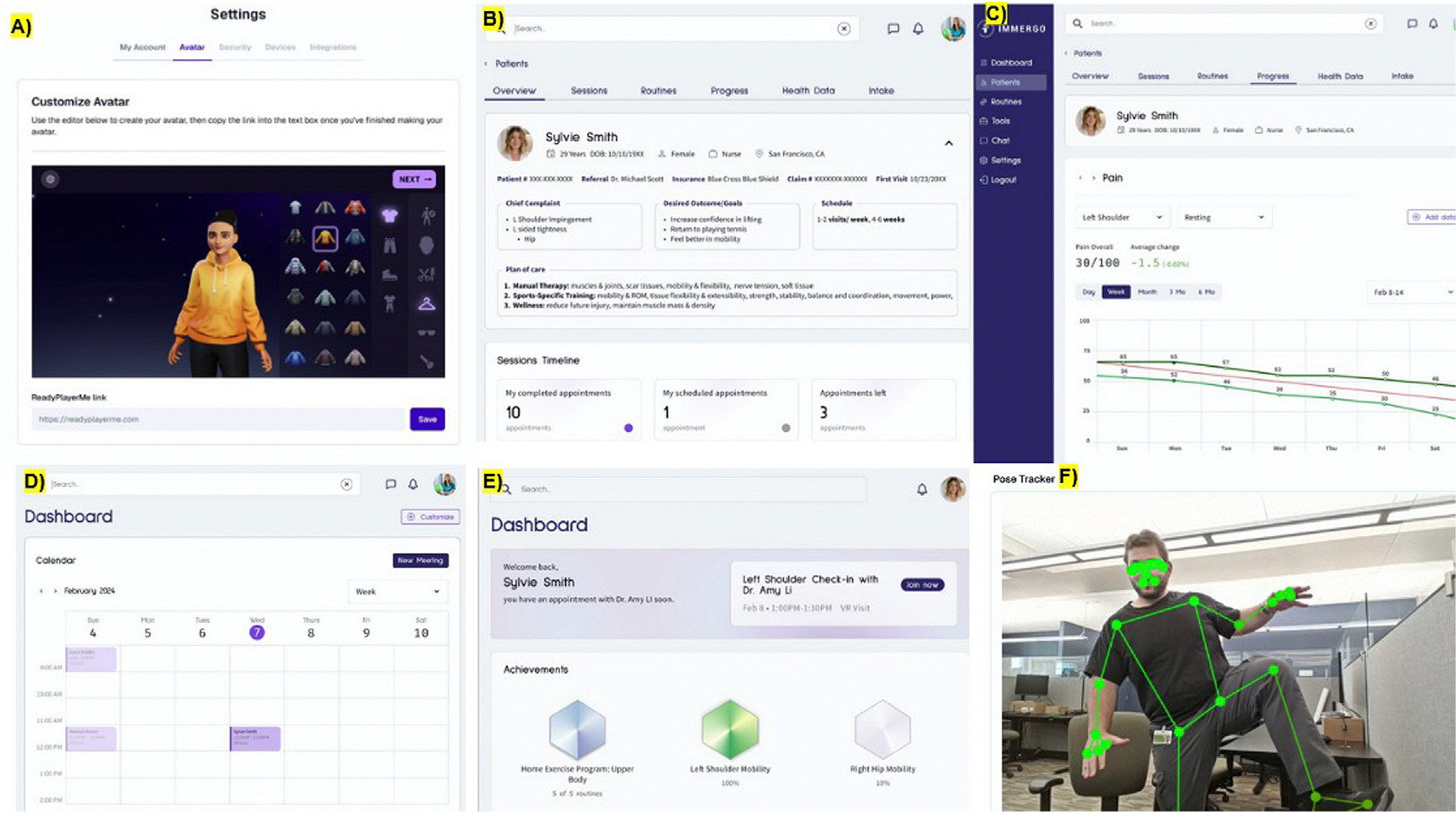
Web Application Prototype Usability Tasks including **(A)** creating an avatar using an embedded *Ready Player Me* window, **(B)** finding and viewing mock patient info, **(C)** viewing mock patient progress, **(D)** reviewing schedule and appointments, **(E)** patient dashboard mock progress and badges, **(F)** enabling full body tracking via web application.

Each focus group was equipped with standardized materials to ensure consistency across sessions:

- VR Hardware: Meta Quest 3 and Meta Quest 2 headsets. Meta Quest devices are standalone VR headsets manufactured by Meta (formerly Facebook) that provide immersive virtual reality experiences without requiring external sensors or cables, enabling accessible VR interaction for rehabilitation applications.
- Documentation Materials: Sticky notes, printed worksheets for single ease-of-use usability rating tasks, four-axis framework analysis templates, and color-coded voting stickers (green for clinicians, red for patients) for prioritization exercises.
- Observation Technology: Laptop displays connected to VR headsets for real-time sharing of individual user experiences with the broader group, enabling collaborative observation and immediate feedback discussion.
- Stimuli and Prototypes: During task exploration and four-axis framework analysis tasks, participants were invited to interact with a variety of VR prototypes (as seen in Figure 2) and Web application prototypes (as seen in Figure 3). VR prototypes included functional embodied avatars, telehealth rooms with real-time data measures, movement recording, and movement playback tooling with placeholder UIs or Figma^3^ prototypes, and web application prototypes included functional full-body pose tracking and placeholder UI elements or Figma prototypes. Avatar creation was implemented using *ReadyPlayerMe*^4^, which provides a web-based interface for generating personalized 3D avatars that can be used across different virtual environments and applications.

Each table was equipped with one Meta Quest 2 headset, one Meta Quest 3 headset, and two laptops or smartphones for casting VR views, enabling group observation. This mixed-hardware approach reflects real-world deployment scenarios in which users access platforms across different device generations. Participants selected headsets based on availability rather than through a systematic assignment process. While the devices differ in visual quality and processing performance, both run identical software with the same interaction methods (controller input schema) and application functionality. The evaluation focused on software design (interface layouts, navigation patterns, interaction workflows, feature organization). Usability tasks assessed participants’ ability to complete application workflows (recording/evaluating movements, creating/completing exercise programs, joining meetings) that remained functionally identical across both devices, with tasks and interaction challenges tied to software design rather than hardware capabilities. The qualitative four-axis framework analysis and stakeholder prioritization exercises, which constituted the primary data collection methods, centered on software interaction paradigms, feature requirements, and interface organization independent of hardware specifications.

### 3.5 Workshop structure

The workshop followed a structured agenda incorporating morning and afternoon sessions with distinct activities designed to progress from individual experience to collaborative design synthesis. The schedule balanced hands-on interaction time with reflective analysis periods, following established co-design workshop frameworks (Antoniou et al., 2024). The full workshop schedule and methodology are detailed in Table 2.

### 3.6 Data collection and analysis

A mixed-methods approach combined qualitative and quantitative data collection methods, prioritizing rich stakeholder feedback while maintaining systematic documentation for evidence-based design decisions (Cucinella et al., 2025):

#### 3.6.1 Qualitative measures

- Four-Axis Framework Worksheets: After exploring and evaluating key Immergo features for both platforms, each group systematically organized their usability insights into four evidence-based categories: Raise (identify features that worked well and to be further emphasized), Reduce (highlight features that were less effective and to be further minimized), Eliminate (pinpoint features to remove), and Create (propose new features to introduce). This framework facilitates critical reflection and structures participant feedback to amplify strengths, address pain points, and identify innovation opportunities (Antoniou et al., 2024). Each group completed one comprehensive worksheet for the web application and one for the VR platform, ensuring systematic coverage of both interaction modalities.
- Stakeholder Priority Synthesis: Features, issues, and ideas derived from the four-axis framework worksheets were written onto individual sticky notes and collaboratively organized on group posters. Through structured group discussion, participants ranked ideas from most important (top of poster) to least important (bottom), creating visual priority hierarchies that reflected consensus stakeholder values. Each group produced one systematic priority cluster representing collective stakeholder perspectives on development priorities.
- Cross-Group Validation: Following individual group priority development, participants engaged in a gallery walk methodology where they reviewed other groups’ priority clusters and used color-coded stickers (green for clinicians, red for patients) to indicate cross-stakeholder validation of ideas. This process enabled the identification of themes with broad stakeholder support versus those reflecting specific subgroup needs.
- Exit Survey Sentiment Evaluation: During the closing session, participants completed comprehensive surveys assessing the overall usability, accessibility, and comfort of both platforms. Clinicians and patients responded to structured open-ended questions regarding the most promising and challenging aspects of Immergo’s telehealth system, their highest-priority feature enhancements, and their impressions of the workshop’s structure and facilitation effectiveness.

#### 3.6.2 Quantitative measures

- Task-Based Usability Ratings: As part of prototype platform exploration, clinicians and patients completed predefined task sequences within the VR platform and web application. After completing each task, participants responded to the prompt: “How easy or difficult was it to complete this task?” using a 5-point Likert scale (1 = very difficult, 2 = difficult, 3 = neutral, 4 = easy, 5 = very easy). This streamlined approach generated comparable usability scores across platforms and user types while maintaining participant focus on collaborative design activities rather than extensive evaluation procedures. The study research team selected tasks representing core user workflows, including exercise recording, progress review, and clinician-patient interaction scenarios.
- Stakeholder Priority Quantification: Following four-axis framework analysis and group priority ranking, cross-group voting with color-coded stickers provided quantitative measures of stakeholder consensus on design priorities. During the gallery walk, each participant received two votes per other group’s poster to allocate as they chose, using color-coded stickers to maintain stakeholder type identification (green for clinicians, red for patients). Participants were not required to use all votes, allowing selective prioritization of themes they viewed as most critical.
- Workshop Satisfaction and Engagement Assessment: Exit surveys included 5-point Likert scale ratings evaluating workshop satisfaction, likelihood of future platform use, comfort with VR technology, and perceived platform relevance to clinical/personal needs. These quantitative measures were complemented by open-ended elaboration opportunities, enabling a comprehensive understanding of stakeholder experiences and research question validation.

### 3.7 Recruitment strategy

Participants were recruited through multiple channels to ensure diverse stakeholder representation and clinical expertise relevant to embodied telehealth development:

- Academic Institution Referrals: Professional networks from collaborating institutions (Baylor College of Medicine, University of Montana) provided access to clinicians with research experience and patients with diverse rehabilitation backgrounds.
- Clinical Professional Networks: Immergo’s existing Clinician Panel, consisting of rehabilitation professionals with prior telehealth and technology experience, contributed domain expertise and real-world clinical perspectives.
- Patient Community Engagement: The Immergo Waitlist, consisting of potential early-access users who had expressed interest in VR rehabilitation, provided patient perspectives from individuals motivated to engage with innovative rehabilitation approaches.

### 3.8 Ethical considerations

This study received Institutional Review Board (IRB) approval under record #IRB00000266 with participating sites including Baylor College of Medicine, Michael E. DeBakey Veterans Affairs Medical Center, University of Montana, and University of California, Santa Cruz. All participants provided informed consent prior to participation and were informed of their right to withdraw at any time without consequences. This research is supported by the National Science Foundation Small Business Innovation Research Program under Grant No. #2304278 with Immergo Labs as the primary awardee and Baylor College of Medicine and the Michael E. DeBakey Veterans Affairs Medical Center as subawardees. Workshop materials used in this study are provided in the Supplementary Appendix.

### 3.9 Data analysis approach

Workshop materials, including presentation content, completed usability worksheets, photographic documentation of priority clusters, and stakeholder feedback synthesis, were systematically compiled for thematic analysis following established qualitative research procedures. Quantitative usability ratings were analyzed using descriptive statistics segmented by user type (clinician versus patient) and platform (VR versus web) to identify differential user experience patterns. Cross-group voting data was compared to identify consensus priorities while maintaining the visibility of stakeholder-specific preferences for balanced development planning. Qualitative data from four-axis framework worksheets and exit surveys underwent systematic thematic analysis to identify recurring patterns, conflicting perspectives, and innovation opportunities. Integrating quantitative and qualitative findings enabled a comprehensive understanding of stakeholder needs while providing evidence-based recommendations for meaningful XR rehabilitation platform development.

## 4 Results

### 4.1 Overview of findings

The stakeholder workshop gathered feedback from 24 participants, including ten clinicians, eight patients, and six product team members across five focus groups. This section presents findings organized by evaluation modality (VR application, web application, cross-platform prioritization, and stakeholder sentiment), followed by systematic analysis through participatory design frameworks. Quantitative usability ratings are reported descriptively to characterize patterns across platforms and user groups; given the sample sizes, we note these measures should be interpreted as formative design insights rather than statistically generalizable outcomes. The results address three interconnected research questions emerging from co-design theory: 1. What usability barriers impede meaningful engagement with embodied XR rehabilitation platforms? 2. How do clinician and patient priorities converge and diverge in shaping requirements for therapeutic technologies? 3. Which design principles emerge as foundational for meaningful XR experiences that support sustained rehabilitation engagement? The findings reveal thematic differences between clinician and patient perspectives on platform usage, with convergent themes emerging around simplified navigation, enhanced clinical assessment capabilities, and adaptive input modalities for diverse user needs. These patterns provide evidence for design principles grounded in embodied cognition (Riva et al., 2019), participatory design (Qi and Yu, 2025), and accessibility frameworks (Gerling and Spiel, 2021).

### 4.2 VR app usability

Participants systematically evaluated early prototypes of the Immergo VR platform (version 0.123) through structured task-based usability testing, providing quantitative ratings and qualitative feedback on interface design, interaction mechanisms, and movement tracking capabilities (Figure 2).

Usability ratings revealed notable differences between user groups (Table 3), with clinicians rating “Evaluate a Movement” as the most challenging task (M = 2.67, SD = 1.63) due to the far field interaction of the prototype VR real-time movement data viewer UI along with all planes of movement data being exposed. At the same time, patients found “Record a Movement” moderately difficult (M = 3.43, SD = 1.13) as the VR prototype was missing some haptic and audio cues. Both groups rated environmental customization highly (clinicians: M = 4.75, SD = 0.46; patients: M = 4.00, SD = 1.41), suggesting that personalization features contribute significantly to user satisfaction in meaningful XR experiences. The large standard deviation for clinician movement evaluation (SD = 1.63) indicates substantial variability in professional assessment capabilities, likely reflecting diverse clinical expertise levels and technological familiarity.

#### 4.2.1 VR positive feedback themes

Stakeholder feedback identified five primary strengths in the VR platform design that contribute to meaningful rehabilitation experiences:

1. Immersive and Customizable Environments Enhance Engagement: Users responded enthusiastically to the ability to switch between clinical and themed environments (Figure 2G), describing them as “cute,” “cool,” or “very fun.” The visual variety may have enhanced the sense of presence as fantastical VR environments greatly differ from traditional rehabilitation settings. This finding may align with meaningful XR, where affording environmental personalization may support engagement beyond momentary interaction.
2. Real-time Movement Tracking Provides Clinical Value: The visualization of joint angles and range of motion (ROM) during real-time movements received positive feedback from both clinicians and patients (Figure 2C). Many expressed a desire for more muscle group engagement and performance measures toward conducting objective assessments in remote rehabilitation. As one clinician noted, “Being able to see the joint angles in real-time helps me understand if the patient is performing the movement correctly.”
3. Gamification Elements Improve Motivation and Adherence: Participants described the system as more enjoyable and motivating than conventional exercises (e.g., resistance bands), with the game-like presentation and feedback mechanisms contributing to enhanced therapy engagement (Figures 2E,F). In considering meaningful XR, this may support sustained behavioral change that emerges from intrinsically motivating experiences rather than pure external compliance measures.
4. Visual Feedback Enhances Understanding and Motivation: Features such as angle measurements, avatar playback, and muscle group highlights were well-received across both user groups. Many users found these tools informative and motivating, with several expressing excitement at seeing their movements visualized in novel ways. This visual feedback may create meaningful connections between virtual actions and real-world therapeutic progress.

#### 4.2.2 VR challenges and design opportunities

Analysis revealed five significant challenges that impede meaningful XR rehabilitation experiences and require systematic attention:

1. Accessibility Challenges in Navigation and Input: Some participants reported difficulty navigating the virtual environment due to the dexterity requirements on the Meta Quest VR controllers. For instance, one user with hand issues could not reliably press interface buttons, and another struggled to navigate the indirect menu prototypes due to ray-cast hand stability challenges. These limitations point to a need for adaptive input systems in future systems, allowing users to interact through direct raycasting and other inputs such as direct hand interaction, voice controls, and eye gaze with pinch confirmation.
2. Over-Reliance on Indirect Interaction with Controllers Detracted Usability in the Prototype: Multiple users struggled with precise controller-based input of raycasting to a far-field user interface and clicking the trigger to select, often triggering unintended features. One participant repeatedly activated a drawing tool by unintentionally pressing the grip button and was confused when they could no longer interact with other UI elements. Another participant wanted customizable button mapping and a more intuitive, possibly hands-free, input method such as hand-tracking and voice controls.
3. Insufficient Multimodal System Feedback Can Create User Uncertainty: Given the nature of some low-fidelity prototypes relying only on visual feedback, several participants were unsure whether the system had successfully registered their actions. For example, one user attempted to save a recorded movement but received no confirmation or feedback, realizing they had not entered a required exercise name. Another suggestion from a participant included introducing a pop-up prompt after pressing “save” to guide users through naming their recordings. For higher fidelity or production systems, it is critical to afford the user multiple stimuli of feedback from visual, audio, and haptic.
4. Unclear Content Design Language: Some terminology and iconography within the prototype application confused some patients and clinicians. For example, the term “evaluate” was unclear to some users, who did not know which features it encompassed or what steps were required. Others struggled to locate previously recorded exercises or differentiate between “record” and “make an exercise program,” leading to task confusion. Co-design discussion and stakeholder alignment were valuable for improving content design language and product concepts.

#### 4.2.3 VR four-axis framework analysis results

Systematic application of the four-axis framework (Raise, Reduce, Eliminate, Create) generated comprehensive stakeholder recommendations organized by design action category. This structured approach enabled the identification of design strengths to amplify, weaknesses to address, and innovation opportunities to pursue.

The four-axis framework applied in this study represents a structured implementation of critical reflection principles in participatory design (Qi and Yu, 2025), where systematic organization of feedback across four action categories (Raise, Reduce, Eliminate, Create) enables stakeholders to move beyond reactive commentary toward strategic design prioritization (Kim, 2005). This structured approach aligns with Experience-Based Co-Design (EBCD) methodologies in healthcare (Donetto et al., 2015), which emphasize systematic capture and organization of lived experience to inform service improvement. By separating features to amplify, minimize, remove, and introduce, the framework facilitates the type of mutual learning between end-users and developers that characterizes effective participatory design processes (Qi and Yu, 2025).

##### 4.2.3.1 VR raise priorities

- Interface Simplification for Cognitive Load Reduction: Both clinicians and patients emphasized restructuring key menus (calendar, assigned exercises) for immediate access upon application launch. This reflects meaningful XR design principles where reduced cognitive burden enables focus on therapeutic activities rather than interface navigation.
- Customization Features for Personalized Engagement: Participants requested adaptive interface elements, including adjustable text sizes, resizable panels, and personalized virtual environments. These customization capabilities support meaningful XR by enabling users to tailor experiences to individual needs and preferences, promoting sustained engagement.

##### 4.2.3.2 VR reduce priorities

- Interface Clutter and Information Overload: Stakeholders recommended streamlining therapy session views to display only contextually relevant data (e.g., shoulder-specific metrics during shoulder exercises).
- Environmental Distractions: While many users appreciated fantasy environments, some clinicians suggested limiting non-clinical virtual environments for telehealth appointments (e.g., “space” or “mountain” settings) that detracted from therapeutic focus, emphasizing the importance of clinically relevant context in meaningful rehabilitation XR. This suggests affording both serious and playful environments for user customization and perhaps defaulting to the more traditional or serious environments as users acclimate to the capabilities afforded in XR.

##### 4.2.3.3 VR create priorities

- Integrated Tutorials and Progressive Onboarding: Despite completing standard VR tutorials, participants with limited XR experience required deeper application-specific guidance for therapeutic workflows. This suggests the need for domain-specific onboarding that bridges general VR literacy with clinical application competency.
- Expanded Clinical Assessment Integration: Clinicians requested additional comprehensive assessment tools, including functional measures (e.g., Forward Reach Test, Timed Up and Go), balance assessments (e.g., Berg Balance Scale), and neurological screening capabilities to match in-person care depth beyond objective measurement biomechanics tools.
- Multimodal Real-time Movement Correction and Safety Feedback: Both user groups emphasized immediate corrective feedback through visual, audio, and haptic cues, animated demonstrations, and verbal prompts to ensure safe, effective exercise performance in remote contexts.

##### 4.2.3.4 VR eliminate priorities

- Non-essential Administrative Features in VR Context: Stakeholders recommended relocating administrative functions (scheduling, billing, revenue tracking) to companion web applications, allowing VR environments to focus exclusively on therapeutic interaction.
- Lack of Adaptive Inputs in the Lofi Prototype: Participants advocated for reducing the prototype’s dependence on handheld controllers, particularly for users with motor impairments or those requiring hands-free interaction during exercise activities. This aligns with the accessibility needs of XR hardware to afford alternative input methods to support users with disabilities (Elor and Ward, 2021; Kurniawan et al., 2024).

### 4.3 Web application usability assessment

Stakeholders evaluated the web-based dashboard and rehabilitation management tools, focusing on information accessibility, workflow integration, and cross-platform coherence with VR components (Figure 3).

Web application usability patterns (Table 4) revealed complementary strengths and challenges compared to VR interaction. Clinicians rated patient findings (M = 4.61, SD = 0.601) and measures review (M = 4.50, SD = 0.707) highly, indicating successful workflow integration for professional tasks. However, avatar creation with Ready Player Me presented challenges for both groups (clinicians: M = 2.83, SD = 1.472; patients: M = 3.57, SD = 0.976), suggesting interface complexity in cross-platform identity management. The high variability in patient progress review (M = 2.86, SD = 1.864) indicates inconsistent user experience design that may impede meaningful engagement with personal health data, suggesting a need for personalized patient progress modules.

#### 4.3.1 Web positive feedback themes

- Clinician Workflow Optimization: The dashboard structure effectively supported patient monitoring and clinical decision-making (Figures 3B,C), with high usability ratings for core professional tasks.
- Asynchronous Progress Review: Patients appreciated the ability to review exercise history and progress metrics outside of active therapy sessions, supporting meaningful engagement with personal health data over time.
- Cross-Platform Communication: The web application facilitated clear clinician-patient communication through integrated messaging and progress-sharing capabilities (Figures 3D,E).

#### 4.3.2 Web challenges and design opportunities

- Information Density and Cognitive Overload: Stakeholders reported overwhelming information presentation that hindered efficient task completion, particularly in progress review interfaces.
- Navigation Complexity: Difficulty locating key performance metrics and functionality suggested suboptimal information architecture for clinical and patient workflows.
- Limited Customization Options: Users requested greater interface personalization to accommodate diverse clinical specializations and patient preferences.

#### 4.3.3 Web application four-axis framework results

Web platform analysis revealed distinct themes compared to VR evaluation, emphasizing traditional computing efficiency and cross-platform integration:

##### 4.3.3.1 Web raise priorities

- Exercise Library Expansion: Clinicians specifically requested broader occupational therapy activities, including hand-eye coordination, fine motor skills, and sensory integration exercises to serve diverse patient populations.

##### 4.3.3.2 Web reduce priorities

- Administrative Information Overload: Participants recommended redistributing complex data (revenue, detailed analytics) across separate interface sections rather than consolidated dashboards.
- Communication Expectation Misalignment: Stakeholders clarified response time expectations (typically two business days) to prevent patient frustration with communication delays.

##### 4.3.3.3 Web create priorities

- Personalized Health Education Integration: Both user groups requested condition-specific educational content, including nutrition guidance, lifestyle modifications, and evidence-based explanations of prescribed exercises.
- Enhanced Clinical Documentation: Clinicians requested comprehensive note-taking capabilities, treatment goal tracking, and external document integration to support continuity of care.
- Advanced Progress Visualization: Stakeholders requested multi-metric displays, goal-setting capabilities, and time-based comparison tools to enhance meaningful engagement with progress data.

##### 4.3.3.4 Web eliminate priorities

- Redundant Interface Elements: Participants identified duplicated features (assignment widgets, calendar displays) that should be consolidated for streamlined navigation.
- Outcome-Based Achievement Systems: Clinicians recommended replacing clinical outcome achievements with participation-based rewards to maintain motivation while focusing on patient-controllable factors.

### 4.4 Cross-platform feature prioritization and stakeholder consensus

Systematic synthesis of four-axis framework results across both platforms generated quantified stakeholder priorities through structured voting exercises (Figure 1C). Cross-platform feature prioritization results are presented in Table 5. This cross-group validation process identified design themes with broad stakeholder support versus those reflecting specific subgroup needs.

The prioritization results reveal clear stakeholder consensus on fundamental design principles for meaningful XR rehabilitation. The top four priorities, minmalist interface and navigation (19 votes), expanded assessment and metrics (17 votes), tutorials/onboarding (9 votes), and customization/personalization (9 votes), collectively represent 50% of the votes indicating strong agreement on core usability and clinical functionality requirements.

#### 4.4.1 High-priority design themes (15+ votes)

1. Minimalist Interface and Navigation (19 votes): The highest-priority theme reflects stakeholder consensus on reducing cognitive load through streamlined menu structures, intuitive information hierarchy, and context-aware interface adaptation. This finding may suggest that meaningful XR design principles should emphasize user agency and reduced friction in therapeutic interactions.
2. Expanded Assessment and Metrics (17 votes): Strong clinical stakeholder support for comprehensive assessment integration demonstrates the critical importance of matching in-person care depth through functional measures, balance assessments, and condition-specific tracking capabilities.

#### 4.4.2 Moderate-priority design themes (5–9 votes)

1. Tutorials, Help, and Onboarding (9 votes): Equal prioritization with customization indicates stakeholder recognition that effective onboarding is essential for meaningful long-term platform engagement.
2. Customization and Personalization (9 votes): Stakeholder emphasis on adaptive interfaces reflects the diverse needs of rehabilitation populations and the importance of user agency in meaningful XR experiences.
3. Clinical Accuracy and Improved Tracking (8 votes): Strong support for enhanced movement tracking validates the critical importance of embodied telepresence accuracy for remote rehabilitation effectiveness.
4. Gamification and Motivation (7 votes): Moderate prioritization suggests stakeholder appreciation for engagement mechanisms while maintaining focus on clinical functionality over entertainment value.
5. Adaptive Inputs and Accessibility Features (6 votes): Consistent prioritization across groups demonstrates recognition of inclusive design requirements for diverse patient populations.

#### 4.4.3 Emerging design themes (1–4 votes)

Lower-frequent themes, including AI integration, job security concerns, and exercise variety, reflect emerging considerations that may gain importance as platform adoption increases and core functionality stabilizes.

### 4.5 Stakeholder sentiment and platform adoption potential

Exit survey responses comprehensively assessed stakeholder attitudes toward VR rehabilitation technology, platform-specific feedback, and adoption likelihood across user groups.

#### 4.5.1 Clinician exit feedback

Clinician exit survey responses (Table 6) revealed cautiously optimistic attitudes toward VR rehabilitation integration, with notable variability in adoption readiness given the prototypes tested and clinical relevance perceptions. Clinician sentiment suggested revealed high adoption interest (M = 4.14, SD = 0.69) despite moderate clinical relevance perceptions (M = 3.13, SD = 0.99), which may suggest enthusiasm for XR technology potential outweighs current platform prototype limitations. The moderate usability rating (M = 3.63, SD = 0.52) with relatively low variability may indicate consistent user experience across diverse clinical expertise levels.

##### 4.5.1.1 Clinician-identified platform strengths

Thematic analysis of open-ended responses revealed two primary value propositions:

1. Enhanced Accessibility and Care Reach (n = 4/8, 50%): Clinicians emphasized VR’s potential to extend rehabilitation services to underserved populations, with one noting, “It can bring PT to individuals that might otherwise have significant difficulty getting to an outpatient appointment.” This accessibility focus aligns with meaningful XR principles of expanding meaningful healthcare access beyond traditional institutional boundaries.
2. Clinical Versatility and Adaptability (n = 3/8, 38%): Clinicians recognized VR’s potential across diverse conditions, including musculoskeletal rehabilitation, chronic pain management, and neurological conditions. As one participant noted, “The broad application of this technology can serve numerous patient populations, including neurological, orthopedic, and chronic pain cases.”

##### 4.5.1.2 Clinician-prioritized feature requirements

When asked to identify the single most important feature improvements, clinicians demonstrated convergent priorities:

1. Movement Tracking Accuracy (n = 3/8, 38%): Nearly half of clinicians emphasized precise body movement capture as essential for clinical validity, noting requirements for whole-body assessment and compensatory pattern identification.
2. Safety and Environmental Awareness (n = 2/8, 25%): Clinicians requested features to ensure patient safety during home-based VR sessions, including environmental hazard detection and fall prevention systems.
3. Enhanced Engagement Mechanisms (n = 2/8, 25%): Remaining clinicians prioritized gamification and environmental customization to improve patient motivation and session completion rates.

##### 4.5.1.3 Clinician-identified implementation barriers

Critical challenges identified by clinicians reflect both technical and usability limitations:

1. Movement Assessment Limitations (n = 3/8, 38%): Clinicians emphasized the inability to view comprehensive body mechanics simultaneously, with one noting, “joints [real-time measurements with the XR prototype] do not move in isolation, and being able to accurately see the legs and torso will be important.”
2. Interface Complexity for Target Populations (n = 3/8, 38%): Equal concern focused on usability barriers for older adults and technologically inexperienced users representing significant rehabilitation patient populations.

#### 4.5.2 Patient experience and adoption readiness

Patient sentiment analysis (Table 7) revealed uniformly positive attitudes toward VR rehabilitation technology, with high comfort levels and adoption likelihood across diverse rehabilitation backgrounds. Patient sentiment analysis revealed uniformly positive attitudes across all measured dimensions, with high comfort (M = 4.50, SD = 0.76), expected therapy improvement (M = 4.50, SD = 0.53), and adoption likelihood (M = 4.25, SD = 0.71). Low standard deviations indicate consistent positive experiences across diverse patient backgrounds and VR experience levels.

##### 4.5.2.1 Patient-identified platform benefits

Patient feedback revealed dual value propositions emphasizing both functional and motivational benefits:

1. Real-time Movement Verification (n = 3/8, 38%): Patients valued visual feedback systems that provided exercise performance confirmation, with one noting such features “give verification” of correct exercise execution in the absence of direct clinician supervision.
2. Enhanced Motivation Through Immersive Engagement (n = 3/8, 38%): Equal emphasis on VR’s novelty and game-like presentation as motivation enhancement, with patients describing the technology as making therapy feel “less like a medical routine and more like an interactive game.”

##### 4.5.2.2 Patient-prioritized feature requirements

Patient feature preferences demonstrated convergent priorities across three key areas:

1. Comprehensive Onboarding and Tutorials (n = 2/8, 25%): Patients emphasized the need for exercise demonstration and performance verification before independent session completion.
2. Interface Simplification (n = 2/8, 25%): Equal prioritization of reduced cognitive load and streamlined exercise access to minimize confusion and maximize therapy focus.
3. Real-time Performance Feedback (n = 2/8, 25%): Continued emphasis on immediate form correction and safety guidance during exercise performance.

##### 4.5.2.3 Patient-identified adoption barriers

Patient concerns reflected practical implementation challenges rather than technology resistance:

1. Technology Cost and Accessibility (n = 1/8, 13%): One patient cited VR headset cost as a barrier to personal adoption, highlighting economic accessibility challenges.
2. Therapy Type Limitations (n = 1/8, 13%): One patient noted that manual therapy requirements (tendon mobilization) could not be replicated in VR, potentially indicating the need for hybrid care models rather than a complete digital health replacement.

#### 4.5.3 Cross-stakeholder convergence and divergence

Comparative analysis of clinician and patient feedback revealed both aligned priorities and distinct professional versus personal perspectives on meaningful VR rehabilitation:

##### 4.5.3.1 Convergent priorities

- Interface Simplification: Both groups prioritized reduced complexity and streamlined navigation
- Real-time Feedback Systems: Shared emphasis on movement tracking and performance verification
- Accessibility and Inclusivity: Recognition of diverse user needs and adaptive design requirements

##### 4.5.3.2 Divergent priorities

- Clinical Depth vs. User Experience: Clinicians emphasized comprehensive assessment capabilities, while patients prioritized ease of use and motivation
- Professional Workflow vs. Personal Engagement: Clinicians focused on documentation and clinical decision support, while patients emphasized entertainment and self-efficacy
- Safety Systems vs. Autonomy: Clinicians requested extensive safety monitoring while patients valued independent exercise capability

These patterns of convergence and divergence reflect distinct but complementary stakeholder perspectives predicted by Stakeholder Theory (Freeman, 2010), which posits that different stakeholder groups prioritize outcomes aligned with their primary interests and constraints. The convergent priorities (interface simplification, real-time feedback) represent shared instrumental concerns, features necessary for any stakeholder to accomplish core tasks. The divergent priorities reflect differing ultimate objectives: clinicians prioritize clinical depth to fulfill professional responsibilities for accurate assessment and evidence-based intervention, while patients prioritize user experience to support sustained engagement and self-efficacy. This distinction aligns with Self-Determination Theory (Ryan and Deci, 2000), which proposes that intrinsic motivation requires satisfaction of three psychological needs: competence (patients’ ease-of-use focus), autonomy (patients’ preference for independent exercise), and relatedness (clinicians’ emphasis on maintaining therapeutic relationships through comprehensive communication tools). The co-design process helped surface these distinct motivational frameworks, enabling design decisions that address instrumental needs universally while providing role-specific pathways for satisfying divergent psychological needs.

### 4.6 Implications for meaningful XR rehabilitation platform development

The stakeholder feedback analysis provides design considerations for developing meaningful XR rehabilitation platforms that create lasting physical health improvements and sustained user engagement. Key findings demonstrate the critical importance of balancing clinical rigor with user experience accessibility, emphasizing adaptive design approaches that accommodate diverse user needs while maintaining therapeutic effectiveness. The convergent priorities across stakeholder groups, including simplified navigation, enhanced clinical assessment, and comprehensive onboarding, provide clear development priorities. The divergent perspectives between clinicians and patients highlight the need for role-specific interface adaptations while maintaining cross-platform coherence for meaningful collaborative rehabilitation experiences. These results establish considerations for XR design in healthcare contexts, demonstrating how systematic co-design processes can identify critical requirements for creating meaningful therapeutic technologies that extend beyond momentary engagement to create lasting health improvements and behavioral change.

In summary, the findings reveal systematic patterns extending beyond the specific platform prototypes. Convergent stakeholder priorities (minimalist interface: 19 votes; clinical assessment: 17 votes) indicate shared foundational requirements, while divergent priorities between clinicians and patients reflect complementary needs requiring simultaneous attention. The accessibility challenges observed during hands-on interaction suggest current default consumer VR hardware may not adequately support rehabilitation populations without adaptive design. These patterns inform the design principles and research implications discussed in the following section. While some findings confirm established XR rehabilitation principles, such as preferences for real-time feedback and gamified environments, this study contributes novel insights specific to embodied telehealth contexts: the strong prioritization of minimalist care interfaces over feature richness among VR novices (78% of participants), the clinician emphasis on comprehensive biomechanical assessment capabilities exceeding current consumer hardware, and the identified tension between accessibility requirements and standard VR controller interaction paradigms. These context-specific findings extend beyond general XR rehabilitation knowledge to inform embodied telehealth design specifically.

## 5 Discussion

This study examined the efficacy of XR experiences in physical rehabilitation environments through the lens of stakeholder co-design. By engaging clinicians, patients, and product teams in structured design activities, we identified high-priority needs for embodied telehealth systems and uncovered important usability challenges and design opportunities. The results offer a robust empirical foundation for co-design methodologies in developing XR rehabilitation platforms, highlighting the delicate balance between technological advancements and the practical requirements of clinical settings.

### 5.1 Design implications for XR rehabilitation developers

The convergent stakeholder priorities and usability challenges identified in this study provide actionable guidance for designers developing XR rehabilitation systems. The clear preference for simplified navigation combined with observed indirect controller interaction difficulties suggests that XR rehabilitation interfaces should prioritize alternative inputs, such as direct manipulation and gesture-based interaction, over traditional pointer ray interaction with controllers. Designers should consider implementing alternative input methods beyond indirect interaction (hand tracking direct touch, gaze and pinch, voice navigation) and progressive disclosure interfaces that present minimal complexity initially while enabling advanced functionality as users develop competency.

The platform-specific usability patterns, with web applications excelling at information management and VR providing a superior immersive interaction, may indicate that effective XR rehabilitation systems require the strategic distribution of functionality across complementary platforms. Rather than attempting to replicate all features within VR environments, designers should leverage each platform’s strengths: web applications for traditional computing tasks such as scheduling, progress review, and detailed 2D data analysis, and VR for embodied or spatial interaction such as movement training, real-time assessment, and immersive therapeutic guidance.

The accessibility challenges encountered during prototype testing highlight critical design requirements for rehabilitation populations. Designers must consider multiple input modalities from the initial design phase. Potential needs include modalities such as voice commands for users with limited dexterity, eye tracking for hands-free navigation, and haptic feedback for users with visual impairments. The frequent unintended controller interactions observed in our study suggest that rehabilitation XR systems may require custom input devices or significantly enhanced gesture recognition capabilities.

These design implications emerge directly from patterns observed during the workshop. The preference for simplified navigation (19 votes, Section 4.4) combined with observed controller interaction difficulties (Section 4.2.2) provides evidence that XR rehabilitation interfaces may require alternative input paradigms. The platform-specific usability patterns, web applications excelling at information management (clinician patient finding: M = 4.61) while VR provided superior immersive interaction (environmental customization: M = 4.75), suggest complementary platform roles rather than feature replication. The accessibility challenges encountered by participants with hand dexterity limitations (Section 4.2.2) reinforces research demonstrating that VR hardware often assumes able-bodied interaction (Gerling and Spiel, 2021), establishing support for multi-modal adaptive input requirements.

### 5.2 Embodied telehealth as a new paradigm for remote healthcare

The stakeholder consensus on full-body tracking accuracy as essential for clinical adoption establishes embodied telepresence as a fundamental requirement for meaningful remote rehabilitation. Unlike traditional telehealth’s audiovisual limitations (Matamala-Gomez et al., 2021), embodied telehealth enables clinicians to observe, assess, and correct patient movement patterns in real-time across physical distances.

Our findings suggest three critical components of effective embodied telepresence for rehabilitation:

1. Biomechanical Fidelity: Accurate capture and representation of joint angles, movement patterns, and compensatory behaviors that clinicians rely on for assessment and intervention planning.
2. Responsive Correction: Real-time feedback systems provide immediate movement guidance when clinicians cannot physically manipulate or guide patient positioning.
3. Shared Presence: Virtual environments that maintain therapeutic relationships through embodied avatar interaction rather than reducing clinicians to disembodied voices or screen presences.

These components operationalize established telepresence theory in healthcare contexts. Steuer’s foundational telepresence framework (Steuer, 1992) defines telepresence as the extent to which individuals feel present in a mediated environment rather than their immediate physical environment, determined by vividness (sensory breadth and depth) and interactivity (speed, range, and mapping of responses). Our findings extend this framework by demonstrating that rehabilitation telepresence requires not only visual and spatial presence but also proprioceptive and kinesthetic presence, the sense that one’s body movements in virtual space correspond accurately to therapeutic intent. This aligns with recent work on embodiment in virtual reality (Kilteni et al., 2012), which establishes that sense of embodiment comprises body ownership (feeling that the virtual body is one’s own), agency (feeling control over the virtual body), and self-location (feeling spatially located within the virtual body). The stakeholder emphasis on full-body tracking accuracy and real-time movement feedback suggests that meaningful embodied telehealth requires high levels of all three embodiment components to support effective therapeutic intervention.

Patient and provider satisfaction for VR meeting environments over traditional videoconferencing in this study and previous (Elor et al., 2022) works suggests potential advantages for embodied interaction in therapeutic relationships. However, these preferences emerged from brief prototype exposure and may not persist through extended clinical use. Longitudinal studies comparing embodied telepresence with traditional telehealth modalities will be essential for validating these preliminary findings.

The integration challenges between VR and web platforms highlight the complexity of designing coherent cross-platform experiences for healthcare contexts. The web application’s strength in information management and the VR platform’s advantages for immersive interaction suggest that effective embodied telehealth may require sophisticated coordination between complementary technologies rather than attempting to replicate all functionality within VR environments.

### 5.3 Balancing immersion and cognitive load in rehabilitation XR

The divergent usability patterns between VR and web platforms reflect fundamental differences in cognitive processing demands. While VR platforms received lower average usability ratings for complex tasks like movement evaluation, web platforms excelled at information-dense activities like patient data review (M = 4.50). This pattern may align with Cognitive Load Theory (Sweller, 2011), which posits that effective learning and task performance depend on managing intrinsic load (task complexity), extraneous load (presentation format), and germane load (schema construction). VR interfaces can inherently introduce extraneous cognitive load through spatial navigation, 3D interaction, and novel input modalities (Makransky and Petersen, 2021), which has a potential to overwhelm users when combined with high intrinsic load tasks like clinical assessment involving multiple biomechanical parameters.

The stakeholder prioritization of minimalist interface design (19 votes) and progressive complexity (implicit in customization requests) reflects an intuitive understanding of split-attention effects (Sweller, 2011), where presenting information across multiple spatial locations or modalities increases cognitive load. The requests to display “only contextually relevant data” (e.g., shoulder-specific metrics during shoulder exercises) demonstrate recognition that effective rehabilitation XR must implement spatial and temporal contiguity principles, presenting related information together in space and time to reduce cognitive integration demands. This finding has critical implications for embodied telehealth design: while full-body tracking provides rich biomechanical data, effective therapeutic interfaces must selectively filter and present information based on current clinical focus to prevent cognitive overload that could impede both clinician assessment and patient understanding of corrective feedback.

### 5.4 Stakeholder co-design consensus

The stakeholder prioritization identified the highest-priority design aspects, with minimalist interface navigation (19 votes) and expanded clinical assessment capabilities (17 votes) emerging as clear priorities. This convergence across diverse stakeholder groups provides valuable validation for focusing development efforts on core usability and clinical functionality rather than advanced features.

However, the moderate priority assigned to adaptive inputs and accessibility features (6 votes) deserves careful consideration, given the significant accessibility challenges identified during hands-on testing. This potential disconnect between voting priorities and observed usage difficulties suggests that accessibility requirements may be underestimated by stakeholders who have not yet experienced the full scope of motor and dexterity challenges that emerge during extended VR use in rehabilitation contexts.

The workshop methodology aligns with core participatory design principles identified across HCI research: sharing power through equal stakeholder representation and voting mechanisms, prioritizing relationships via facilitated group dialogue, using participatory means through hands-on prototype interaction, and building capacity by engaging diverse expertise levels (Qi and Yu, 2025). This principled approach addresses a critical gap identified in healthcare XR development, where Antoniou et al. (Antoniou et al., 2024) emphasize that authentic stakeholder engagement, organizational rigor, and face-to-face collaboration are essential for translating user requirements into technical specifications. The convergent priorities across diverse stakeholder groups validate the effectiveness of this structured participatory approach for identifying fundamental design requirements rather than superficial preferences.

### 5.5 Contributions to meaningful XR in healthcare

Our results suggest meaningful XR frameworks (Riva et al., 2019) that demonstrate how embodied telepresence can create authentic therapeutic relationships in remote contexts. The finding that stakeholders prioritized real-time movement tracking and corrective feedback (17 votes for expanded assessment capabilities, Section 4.4) provides evidence supporting Riva et al.’s embodied cognition framework, where VR’s effectiveness stems from shared neural mechanisms between virtual and physical experiences. Unlike traditional telehealth that reduced therapists to feeling like only “life coaches” (Elor et al., 2022), the stakeholder feedback (Section 4.5) suggests that embodied XR interactions may maintain clinical rigor through accurate movement capture and immediate corrective guidance. Specifically, 50% of clinicians identified enhanced accessibility and care reach as primary benefits, while 38% of patients valued real-time movement verification.

This embodiment-driven approach aligns with the Cognitive Affective Model of Immersive Learning (CAMIL) (Makransky and Petersen, 2021), which proposes that immersion and interactivity increase presence and agency, leading to enhanced embodiment that supports behavioral change. However, our findings reveal a critical tension: while embodiment theory suggests high-fidelity body tracking may enhance therapeutic outcomes, 78% of participants were VR novices requiring simplified interfaces. The stakeholder prioritization of minimalist design (19 votes) alongside customization requests (9 votes) suggests that meaningful rehabilitation XR may need to implement progressive embodiment (Gall et al., 2021), where users gradually develop competency through scaffolded complexity rather than immediate exposure to full biomechanical fidelity. This operationalizes the accessibility, expertise tradeoff identified in participatory design literature (Qi and Yu, 2025), providing quantitative support for progressive disclosure approaches that extend previous co-design studies (Muñoz et al., 2022; Shah et al., 2023).

The convergent emphasis on eliminating non-clinical features while expanding assessment capabilities (Sections 4.2.3, 4.3.3) suggests that meaningful healthcare XR may prioritize therapeutic authenticity over entertainment value, contrasting with consumer VR design paradigms. This indicates that rehabilitation contexts may require design principles different from those of entertainment or educational applications.

### 5.6 Technology development and implementation considerations

The frequent VR controller-related usability issues and stakeholder requests for alternative input methods indicate that meaningful rehabilitation XR requires hardware innovations beyond current consumer VR platforms. The specific challenges with indirect precision ray cast interaction and unintended button activation suggest that rehabilitation applications may need specialized input devices or significantly enhanced hand-tracking capabilities. The focus on multimodal feedback, encompassing visual, auditory, and haptic elements, underscores the understanding that practical rehabilitation guidance relies on multiple sensory channels, especially when clinicians cannot offer direct physical support. However, the challenge lies in integrating comprehensive multimodal feedback while ensuring the system remains intuitive and does not overwhelm users with excessive sensory information.

The tracking accuracy requirements identified by clinicians, including whole-body assessment and compensatory pattern detection, may exceed the capabilities of current default consumer VR systems. As with Immergo’s platform, which uses a web platform to stream full-body computer vision key points, clinical-grade movement analysis may require specialized wearable movement trackers or hybrid computer vision systems that combine VR immersion with additional motion capture technologies. The strong clinician interest in VR technology adoption (M = 4.14) despite moderate perceptions of current clinical relevance (M = 3.13) suggests enthusiasm for the potential of immersive technologies while acknowledging the current limitations of the prototypes they experienced in the study. This pattern may indicate that successful clinical adoption will require significant additional development to bridge the gap between technological promise and clinical utility.

### 5.7 Design recommendations for meaningful XR rehabilitation platforms

Based on our stakeholder feedback analysis, we propose six design principles for creating meaningful embodied XR experiences in physical rehabilitation contexts:

#### 5.7.1 Principle 1: embodied guidance through real-time feedback

Unlike 2D interfaces, meaningful VR rehabilitation may leverage embodied interaction for immediate guidance and safety feedback (Riva et al., 2019). Participants emphasized body-centered corrective cues beyond visual instructions, such as embodied avatars highlighting incorrect forms or automated pause-and-correct tutorials interrupting unsafe movements. These embodied features may be essential when clinicians cannot physically intervene, representing a unique value proposition of meaningful rehabilitation XR that can be difficult to replicate in traditional telehealth modalities. This aligns with embodied cognition principles suggesting that multimodal sensory feedback strengthens motor learning and skill transfer (Kilteni et al., 2012; Steuer, 1992).

#### 5.7.2 Principle 2: progressive complexity with minimalist defaults

The tension between novice accessibility and expert customization may require adaptive interface architectures. Platforms could default to minimalist presentations, such as single-joint focus for initial users, while affording options for comprehensive multi-metric dashboards for experienced clinicians depending on provider needs. This progressive disclosure approach may support meaningful long-term engagement by allowing users to discover advanced functionality as competency develops, potentially aligning with scaffolding principles where support structures are gradually removed as learners gain independence (Vygotsky, 1978; Wood et al., 1976). This approach suggests meaningful XR principles of sustained rather than momentary interaction (Riva et al., 2019).

#### 5.7.3 Principle 3: adaptive accessibility through multi-modal input

Meaningful rehabilitation XR may need to accommodate diverse motor capabilities through adaptive input methods, such as hand tracking, face tracking, gaze-based navigation, and voice commands. Research suggests VR’s embodied nature can create barriers for users whose bodies differ from assumed standards (Gerling and Spiel, 2021; Elor and Ward, 2021; Kurniawan et al., 2024). The frequent stakeholder concerns about controller limitations during equipment-based exercises (resistance bands, weights) suggest that meaningful accessibility may require moving beyond standard VR interaction paradigms to support the full spectrum of rehabilitation activities and movement capabilities. Implementing multiple concurrent input modalities could allow users to fluidly switch based on activity demands and individual capabilities, reflecting inclusive design principles (Kurniawan et al., 2024).

#### 5.7.4 Principle 4: clinical authenticity through domain-specific assessment

Rather than generic evaluation tools, meaningful rehabilitation XR may benefit from supporting specialized assessment batteries tailored to clinical subspecialties. The clinician requests for stroke-specific, orthopedic-specific, and neurological assessment tools suggest that meaningful clinical adoption may require deep domain integration rather than broad generalization. This aligns with co-design principles emphasizing that healthcare technologies must be grounded in actual clinical workflows and assessment practices (Cucinella et al., 2025; Duval et al., 2022). Platforms could enable assessment customization along with clinician authoring while maintaining standardized measurement validity and consider their initial user populations when prioritizing assessment capabilities.

#### 5.7.5 Principle 5: biomechanical precision for trust and safety

High-fidelity, real-time movement tracking may represent a foundation of meaningful rehabilitation XR. Clinicians’ emphasis on comprehensive body mechanics assessment and patient reliance on movement verification suggest tracking accuracy functions as both a technical requirement and a trust-building mechanism. Research on embodied virtual reality suggests that accurate visuomotor mapping enhances sense of embodiment and presence (Kilteni et al., 2012), which may be critical for meaningful therapeutic outcomes (Riva et al., 2019). Platforms could prioritize skeletal fidelity and minimize latency while providing transparency about tracking limitations and confidence intervals.

#### 5.7.6 Principle 6: contextual onboarding for therapeutic competency

Meaningful rehabilitation XR may require domain-specific onboarding that builds therapeutic competency rather than general VR literacy. Role-specific tutorials (clinician vs. patient), progressive skill-building exercises, and context-sensitive guidance could enable users to develop meaningful interactions with rehabilitation content rather than simply learning technology operations. This approach aligns with participatory design principles emphasizing that technology training must address domain-specific knowledge and workflows rather than generic technical skills (Qi and Yu, 2025; Antoniou et al., 2024). This principle extends beyond general VR onboarding to address therapeutic contexts’ unique knowledge and skill requirements.

These six design principles collectively inform a framework for Meaningful Embodied Telehealth that may integrate embodied cognition (Riva et al., 2019), cognitive accessibility, universal design (Gerling and Spiel, 2021), and participatory design paradigms (Qi and Yu, 2025). The findings suggest that meaningful rehabilitation XR may require simultaneous attention to multiple design dimensions: embodied fidelity (accurate biomechanical tracking), cognitive accessibility (appropriate information density), inclusive interaction (multi-modal input), and participatory validity (stakeholder-driven requirements). These principles offer considerations for XR rehabilitation platform development, though validation through longitudinal studies with diverse patient populations remains necessary to establish their effectiveness across varied clinical contexts (Greenhalgh et al., 2017).

### 5.8 Toward a research agenda for embodied telehealth

The findings and design principles presented above suggest several critical research directions for advancing meaningful XR in rehabilitation contexts. First, longitudinal validation studies are needed to assess whether platforms implementing these principles achieve superior clinical outcomes, sustained patient engagement, and reduced clinician burden compared to traditional telehealth modalities. Such studies could employ frameworks like NASSS (Greenhalgh et al., 2017) to understand organizational and systemic factors affecting adoption beyond individual usability. Second, comparative effectiveness research could examine which combinations of design principles (e.g., embodied guidance plus adaptive accessibility versus progressive complexity plus clinical authenticity) produce optimal outcomes for specific rehabilitation populations and conditions. Third, accessibility research should investigate how emerging input modalities (eye tracking, EMG-based gesture recognition, brain-computer interfaces) could expand meaningful XR rehabilitation access to populations currently excluded by motor or sensory requirements. Fourth, implementation science studies are needed to understand how embodied telehealth platforms integrate into existing clinical workflows, reimbursement structures, and regulatory frameworks. Finally, cross-cultural validation studies should examine how meaningful XR requirements differ across healthcare systems, cultural contexts, and linguistic groups. This research agenda may extend beyond technology development to encompass the sociotechnical systems necessary for meaningful embodied telehealth to achieve widespread clinical impact.

### 5.9 Limitations and future research directions

While the findings presented above suggest design principles and theoretical implications for meaningful XR rehabilitation, several methodological limitations constrain generalizability and indicate potential directions for future validation research. These limitations are inherent to early-stage participatory design studies prioritizing depth of stakeholder engagement over breadth of generalization (Eisapour et al., 2018; Qi and Yu, 2025), and understanding them clarifies appropriate applications of these findings while highlighting critical next steps for the research community.

Firstly, the single-day workshop format and evaluation of early-stage prototypes yield valuable insights, but these do not replace the need for longitudinal studies utilizing production-quality systems. The predominantly California-based and English-speaking participant pool may also restrict the ability to generalize findings across various cultural and linguistic contexts. This limitation underscores the necessity for cross-cultural validation studies to explore how meaningful XR requirements might differ across diverse healthcare systems.

Additionally, this study employed a qualitative co-design methodology, incorporating descriptive quantitative measures, to provide deeper context on stakeholder feedback. The sample size (N = 10 clinicians, N = 8 patients for usability ratings) was designed to generate rich qualitative insights through multi-stakeholder co-design workshops (Muñoz et al., 2022) rather than for statistical inference or generalizability testing. Usability ratings provided structured feedback prompts and comparative context across platforms and user groups, but were analyzed descriptively without inferential statistical testing. It is important to note this study makes no claims of statistical significance, and findings should be interpreted as formative design insights from an expert stakeholder sample rather than high sample quantitative outcomes. Future validation studies can expand upon this work by using larger samples and controlled conditions to establish statistically significant relationships between design features and usability outcomes.

Moreover, working with early-stage functional prototypes means that specific usability issues identified may stem from temporary implementation constraints rather than fundamental design flaws, particularly when comparing the VR and web application Figma prototypes. Nevertheless, the systematic and convergent feedback from stakeholders suggests that the core findings likely reflect significant requirements rather than limitations specific to any platform. Future research should build on these results through longitudinal evaluations that assess how user needs and system effectiveness evolve, allowing for insights into long-term usability patterns and clinical outcomes rather than just single-session perceptions.

It is also crucial to recognize that most of the participants in this study were VR novices, with 78% indicating that they had limited experience. While this demographic may resemble the early adopter group for clinical XR systems, their feedback may tend to prioritize simplicity. Future studies should investigate how requirements shift as VR literacy improves and users gain more extended exposure to the system.

Several critical research pathways emerge from these limitations. Although stakeholder feedback indicates positive attitudes towards technology, it is essential to empirically validate clinical effectiveness beyond qualitative observations. Implementing research based on frameworks such as NASSS (Greenhalgh et al., 2017) can help identify organizational and systemic factors that facilitate or hinder meaningful adoption of XR in real-world healthcare settings. This approach would address the technical and workflow integration challenges highlighted in this study by focusing on sociotechnical issues beyond individual user acceptance. Lastly, stakeholders’ emphasis on hands-free interaction and adaptive input modalities points to the need for hardware innovations that extend beyond current consumer VR platforms for effective rehabilitation XR. Investigating specialized rehabilitation hardware, adaptive interface technologies, and accessibility-focused input devices could help mitigate existing limitations and broaden the accessibility of meaningful XR, potentially reconciling the gap between prototype capabilities and clinical requirements for biomechanical precision and therapeutic authenticity.

Limitations aside, this study offers a unique exploration into meaningful XR design in healthcare towards embodied telehealth, reinforcing the importance of incorporating stakeholder design features when developing effective embodied telehealth solutions for physical rehabilitation.

## 6 Conclusion

This study showcases how meaningful XR experiences in physical rehabilitation can be systematically designed through comprehensive stakeholder engagement, user-centered co-design methodologies, and careful attention to the unique requirements of embodied telepresence in healthcare contexts. The convergent stakeholder priorities across clinician and patient groups can inspire development roadmaps for creating VR rehabilitation platforms that extend beyond momentary engagement to produce lasting therapeutic benefits and sustained behavioral change. The successful application of structured co-design processes provides a replicable methodology for meaningful XR development across healthcare domains. By prioritizing clinical authenticity, therapeutic safety, and adaptive accessibility, meaningful XR platforms can address the fundamental limitations of traditional telehealth while expanding access to specialized rehabilitation care. As XR technologies continue to mature and healthcare systems increasingly adopt remote care modalities, the principles, and methodologies established in this study can provide essential foundations for developing meaningful experiences that serve both clinical effectiveness and user engagement requirements. The future of XR in healthcare depends not on technological sophistication alone but on systematic understanding and implementation of meaningful experience design that creates lasting value for providers and patients.

## Supplementary Material

VR 4 Axis Framework

Webapp-Clinician

Moderator Notes Template

Appendix Materials Master Excel Sheet

Webapp- Patient

VR App- Clinician

Webapp 4 Axis Framework

Importance Prioritization

VR App- Patient

## Figures and Tables

**TABLE 1:** Participant demographics and experience distribution from immergo stakeholder workshop survey the pre-workshop survey.

Co-design role	N
Clinicians	10
Patients	8
Profession	N
Physical therapist	7
Occupational therapist	1
Clinician researcher	2
Patient	8
Age	%
18–24	11%
25–34	17%
35–44	39%
45–54	17%
55–64	11%
65+	6%
Gender	%
Male	42%
Female	59%
Other	0%
VR experience	%
None	39%
Limited	22%
Moderate	17%
Experienced	11%
Highly exp.	11%
Video call exp.	%
None	0%
Limited	0%
Moderate	11%
Experienced	28%
Highly exp.	61%
Clinician areas	%
Orthopedic	67%
Geriatric	67%
Neurological	44%
Sports	44%
Preventative	44%
Cardiopulm	22%
Clinician areas	%
Pediatric	22%
Patient care	%
Injury recovery	78%
Surgical recovery	33%
Chronic condition	22%
Preventative	0%

Co-Design Role shows clinician vs. patient distribution. Profession details clinician specializations. Age and Gender represent basic demographics. VR, Experience and Video Call Experience use 5-point scales from “Never used” to “Highly experienced.” Treatment Areas shows clinician-patient types (multiple selections). Patient Care shows primary physical rehab reasons (multiple selections).

**TABLE 2:** Co-design workshop schedule and methodology.

Duration	Focus area	Activities
30 min	Introductions and icebreakers	Participant introductions, role identification, and rapport building to establish collaborative workshop environment and surface individual expertise
30 min	Workshop orientation and product overview	Presentation of workshop objectives, agenda, and current immergo platform capabilities to contextualize design challenges and establish shared understanding
45 min	User experience evaluation and design ideation	Hands-on meta quest tutorial and initial usability assessment using streamlined evaluation approach to identify interaction patterns and accessibility barriers
15 min	Break	Rest and informal discussion break
60 min	VR platform task exploration	Assessment of immergo VR platform features through guided task completion with structured feedback collection using 5-point likert ease-of-use ratings
60 min	Extended break	Food break and informal cross-group discussion period
60 min	VR application four-axis framework analysis	Structured evaluation using raise, reduce, eliminate, create framework to systematically identify platform strengths, weaknesses, and innovation opportunities through collaborative reflection
45 min	Web platform task exploration	Assessment of immergo web application functionality through guided task scenarios with comparative ease-of-use rating documentation
30 min	Web application four-axis framework analysis	Application of systematic evaluation framework to a web platform for identifying optimization and enhancement opportunities focusing on workflow integration
15 min	Break	Rest and discussion break
45 min	Stakeholder priority assessment	Individual ranking exercise organizing four-axis insights into priority hierarchy through group discussion and consensus building
30 min	Collaborative priority consensus	Gallery walk methodology for cross-team idea sharing and consensus building on development priorities through structured voting with color-coded stakeholder identification
15 min	Session synthesis	Workshop summary, key findings recap, next steps discussion, and exit survey completion

**TABLE 3:** VR App Usability Ratings by Clinicians and Patients (scale from 1–5 where 1 is very difficult and 5 is very easy).

Clinician usability [N = 10]			Patient usability [N = 8]		
Task	Mean	±SD	Task	Mean	±SD
Record a movement	3.50	0.54	Record a movement	3.43	1.13
Evaluate a movement	2.67	1.63	Playback your movement	4.00	1.00
Create an exercise program	4.00	0.63	Review your progress	4.29	1.11
Change the virtual world	4.75	0.46	Change the virtual world	4.00	1.41
Join a meeting room	3.57	1.62	Join a meeting room	4.29	0.95

**TABLE 4:** Web App Usability Ratings by Clinicians and Patients (scale from 1–5 where 1 is very difficult and 5 is very easy).

Clinician usability [N = 10]			Patient usability [N = 8]		
Task	Mean	±SD	Task	Mean	±SD
Task 1: Create your avatar	2.83	1.472	Task 1: Create your avatar	3.57	0.976
Task 2: Find your patient	4.61	0.601	Task 2: Find your therapist	3.86	1.345
Task 3: Review patient measures	4.50	0.707	Task 3: Review your progress	2.86	1.864
Task 4: Review appointment	3.81	0.753	Task 4: Update your settings	4.83	0.408
Task 5: Enable full body tracking	3.93	0.607	Task 5: Enable full body tracking	3.71	1.380

**TABLE 5:** Cross-platform feature prioritization results based on stakeholder voting themes of non-product team participants.

Theme (higher count = more importance)	Count
Minimalist interface and navigation	19
Expanded assessment and metrics	17
Tutorials, help and onboarding	9
Customization and personalization	9
Clinical accuracy and improved tracking	8
Gamification and motivation	7
Adaptive inputs and accessibility features	6
Provider tools and documentation	6
Therapist workflow options	5
Chat and messaging	4
Web and VR cohesion	3
Safety considerations	2
Exercise library and variability	2
AI replacement concerns	1
AI and voice integration	1

**TABLE 6:** Exit Survey Reflection Clinician Responses [N = 8] (note two clinicians did not complete exit surveys).

Exit question	Mean	±SD
How relevant do you believe the immergo VR platform is to your clinical practice? [1 = not relevant, 4 = highly relevant]	3.13	0.99
How user-friendly did you find the immergo VR platform? [1 = very difficult, 5 = very easy]	3.63	0.52
How likely are you to incorporate VR technology into your practice? [1 = very unlikely, 5 = very likely]	4.14	0.69

**TABLE 7:** Exit survey reflection patient responses [N = 8].

Question	Mean	±SD
How comfortable did you feel using the immergo VR platform? [1 = very uncomfortable, 5 = very comfortable]	4.50	0.76
How do you think the immergo platform could impact your physical therapy experience? [1 = significantly worsen, 5 = significantly improve]	4.50	0.53
How likely are you to continue using VR as part of your physical therapy? [1 = very unlikely, 5 = very likely]	4.25	0.71

## Data Availability

Anonymized workshop data and or materials supporting this study are available from the corresponding author (aviv@immergolabs.com) upon reasonable request, subject to approval from Immergo Labs and institutional review board requirements. Data sharing will comply with participant consent agreements and exclude any potentially identifiable information. Requests should specify the intended use and analysis plan for the requested data.

## Footnotes

1
www.immergolabs.com

2 Fogarty Innovation is a USA 501(c)(3) educational nonprofit organization focused on supporting innovation in medical advancement. More information at https://www.fogartyinnovation.org/

3 Figma Prototypes are interactional functional web tools to test high-fidelity interfaces without code. More information here: https://www.figma.com/prototyping/

4 More information on ReadyPlayerMe at https://readyplayer.me/

## Appendix

Workshop materials used in this study are publicly available at: https://tinyurl.com/elor2025-codesign-xr-appendix.
