# Supplementary material for: Co-designing meaningful extended reality for physical rehabilitation: a stakeholder-driven approach to embodied telehealth
Source: Front Virtual Real. Author manuscript; Available in PMC 2026 Aug 8. (PMC13450176; doi:10.3389/frvir.2025.1675309)
Supplement: Supplementary file 1 [file NIHMS2176626-supplement-Appendix_Materials_Master_Excel_Sheet.pdf]

Artifact	Link	Print Amount	Usage	
Clincian VR Usability Tasks	https://docs.goog	Print 18 (3x6)	Given to clinicans during VR eval	
Patient VR Usability Tasks	https://docs.goog	Print 18 (3x6)	Given to patients during VR eval	
Clincian Web Usability Tasks	https://docs.goog	Print 18 (3x6)	Given to clinicans during webapp eval	
Patient Web Usability Tasks	https://docs.goog	Print 18 (3x6)	Given to patients during webapp eval	
Importance Prioritization	https://docs.goog	30	Given to stakeholders during importance prioritzation exercise	
Moderator Notes Template	https://docs.goog	N/A	Used for moderators to take notes	
4-axis framework VR sheet	https://docs.goog	30	Given to participants after VR evaluation	
4-axis framework Webapp she	https://docs.goog	30	Given to participants after Webapp evaluation	
Sticky Note Pads	Purchase	Purchase	Purchase	(Clinicians get red, Patients get green, Immergo team gets purple)
Voting Stickers	Purchase	Purchase	Purchase	(Clinicians get red, Patients get green, Immergo team gets purple)
Pens	Purchase	Purchase	Purchase	(Clinicians get red, Patients get green, Immergo team gets purple)
Name Tags	Purchase	Purchase	Purchase	(Clinicians get red, Patients get green, Immergo team gets purple)

Time	Focus Area	Activities
AM (~3 hrs)		
9:00am-9:30am (30 mins)	Introductions and Icebreakers	Welcome, introductions, and icebreakers
9:30am-10:00am (30 mins)	Workshop Kickoff and Product Framing	Overview of workshop goals and agenda. Intro to Immergo's current products and design challenges.
10:00am-10:45am (45 mins)	Setting Intentions and Design Thinking Warm Ups	Jump into Meta Quest first steps new user tutorial and warm up with usability and four-actions evaluation framework
10:45am-11:00am (15 mins)	Break	
11:00am-12:00pm (1 hr)	VR App Testing Part 1 - Task Exploration	Running through a variety of tasks on the Immergo VR Platform and sharing feedback around experience.
12:00pm-1:00pm (1 hr)	Lunch!!!	Time for noms
PM (~4 hrs)		
1:00pm-2:00pm (1 hr)	VR App Part 2 - Four Actions Framework Evaluation	4 Actions Analysis - Raise: Identify what worked well and should be emphasized more., Reduce: Identify what didn't work well and should be minimized., Eliminate: Identify what should be completely removed, Create: Identify what should be newly introduced.
2:00pm-2:45pm (45 mins)	Web App Part 1 - Task Exploration	Running through a variety of tasks on the Immergo web application and sharing feedback around experience.
2:45pm-3:15pm (30 mins)	Web App Part 2 - Four Actions Framework	4 Actions Analysis - Raise: Identify what worked well and should be emphasized more., Reduce: Identify what didn't work well and should be minimized., Eliminate: Identify what should be completely removed, Create: Identify what should be newly introduced.
3:15pm-3:30pm (15 mins)	Break	
3:30pm-4:15pm (45 mins)	Importance Prioritization	Each team individually ranks top categories from the 4 axis framework by most important to least important and places on sticky notes.
4:15pm-4:45pm (30 mins)	Importance Voting	Gallery style walkabout to learn about other teams idea's and vote on which two top concepts matter the most to you per each team
4:45pm-5:00pm (15 mins)	Closing	Recap and themes

**Roles**	**Team 1**	**Team 2**	**Team 3**	**Team 4**	**Team 5**
Moderator	Moderator	Moderator	Moderator	Moderator	Moderator
Notetaker	Notetaker	Notetaker	Notetaker	Notetaker	Notetaker
Stakeholder 1	Stakeholder	Stakeholder	Stakeholder	Stakeholder	Stakeholder
Stakeholder 2	Stakeholder	Stakeholder	Stakeholder	Stakeholder	Stakeholder
Stakeholder 3	Stakeholder	Stakeholder	Stakeholder	Stakeholder	Stakeholder
Stakeholder 4	Stakeholder	Stakeholder	Stakeholder	Stakeholder	Stakeholder

Roles					
Faciliator		Head Faciliator			
Moderator					
Notaker					
Clinician Experience					
Patient Experience					
