# Supplementary material for: Co-designing meaningful extended reality for physical rehabilitation: a stakeholder-driven approach to embodied telehealth
Source: Front Virtual Real. Author manuscript; Available in PMC 2026 Aug 8. (PMC13450176; doi:10.3389/frvir.2025.1675309)
Supplement: Supplementary file 2 [file NIHMS2176626-supplement-Importance_Prioritization.pdf]

Immergo Feature Importance Prioritization

Please list the features, issues, or ideas discussed during the workshop in order of importance. Rank them from most important to least important.

Most Important

Least Important

After finishing our list, draw a line on where you think the minimum bar should be to use this platform.

Place any ‘parking lot’ ideas below that you want to be remembered but are not necessarily the most relevant to the immediate app feedback.
