# Supplementary material for: Co-designing meaningful extended reality for physical rehabilitation: a stakeholder-driven approach to embodied telehealth
Source: Front Virtual Real. Author manuscript; Available in PMC 2026 Aug 8. (PMC13450176; doi:10.3389/frvir.2025.1675309)
Supplement: Supplementary file 3 [file NIHMS2176626-supplement-Moderator_Notes_Template.pdf]

Embodied XR Co-Design Workshop Moderator Notes

Date: MM/DD/YYYY

Moderator: Name

Group: (Patients / Clinicians)

Number of Participants:

Media Upload: <link>

Best Practices for Managing Focus Groups

1. **Introduce a Comfortable Environment:**
   - Start with a brief introduction and explain the purpose of the session.
   - Encourage open and honest feedback; remind participants that all opinions are valuable.
   - Ensure that everyone has an opportunity to speak—actively invite quieter participants to share their thoughts.
   - Please remind participants to keep their phones away or turned off during the sessions.
2. **Be Neutral and Non-Judgmental:**
   - Avoid leading questions; ask open-ended questions to elicit more in-depth responses.
   - Stay neutral, even if you disagree with a participant's opinion. Your role is to facilitate, not to influence the discussion.
3. **Keep the Group Focused:**
   - Stick to the agenda, but be flexible if an important issue arises.
   - Gently steer the conversation back on track if it drifts too far from the topic.
   - Manage time effectively to ensure all tasks and discussions are completed.
4. **Encourage Participation:**
   - Use prompts or follow-up questions to draw out more detailed responses.
   - Validate participant input by acknowledging their contributions, which can encourage others to share.
   - Be aware of group dynamics; ensure no single participant dominates the conversation.
   - You are welcome to contribute as well to spark conversation too, but try to prioritize feedback from participants!
5. **Document Key Insights:**
   - Take clear, concise notes on key points, observations, and consensus areas.
   - Capture quotes or specific feedback that might be particularly insightful or illustrative.

    - Note any non-verbal cues or reactions that may indicate strong opinions or feelings.
6. **Close on a Positive Note:**
    - Summarize the key points discussed and thank participants for their time and input.
    - Inform participants about next steps, including how their feedback will be used.

---

0. Intro and Warm Ups

Objective:
Warm stakeholders up and begin identifying design value themes.

Key Tasks Observed:
- Task 1: Intro and how we're feeling
- Task 2: Design value themes

Observations:
- How we're feeling today (record stakeholder one-word feels)
    - ______________________________
    - ______________________________
- Design Values (record stakeholder favorite products and values)
    - ______________________________
    - ______________________________

0.5 First Steps

Objective:
Warm stakeholders up and begin identifying design value themes.

Key Tasks Observed:
- First Steps Demo

Key Discussion Points:

- **Increase:**
    - ______________________________
    - ______________________________
- **Reduce:**
    - ______________________________
    - ______________________________
- **Introduce:**
    - ______________________________
    - ______________________________
- **Eliminate:**

- ○ ____________________
- ○ ____________________

General Insights:

- ____________________
- ____________________

---

1. VR App Usability Testing

Objective:
Evaluate the ease of use, functionality, and user experience of the VR app.

Key Tasks Observed:

- Task 1: Record an Exercise
- Task 2: Introduce and Play an Exercise Program
- Task 3: Evaluate Movement Range of Motion
- Task 4: Change the Virtual Environment
- Additional tasks:

Observations:

- **General Feedback:**
  - ____________________
  - ____________________
- **Challenges Noted:**
  - ____________________
  - ____________________
- **Positive Reactions:**
  - ____________________
  - ____________________
- **Suggestions for Improvement:**
  - ____________________
  - ____________________

---

2. VR App 4-Axis Framework (Increase, Reduce, Introduce, Eliminate)

Objective:
Identify specific areas of the VR app to amplify, simplify, add, or remove.

Key Discussion Points:

- **Increase:**
    - ______
    - ______
- **Reduce:**
    - ______
    - ______
- **Introduce:**
    - ______
    - ______
- **Eliminate:**
    - ______
    - ______

General Insights:

- ______
- ______

---

3. Web App Usability Testing

Objective:
Evaluate the usability, functionality, and user experience of the web app.

Key Tasks Observed:

- Task 1: Review Patient Data
- Task 2: Schedule a Session
- Task 3: Enable Full Body Tracking
- Additional tasks:

Observations:

- **General Feedback:**
    - ______
    - ______
- **Challenges Noted:**
    - ______
    - ______
- **Positive Reactions:**
    - ______
    - ______
- **Suggestions for Improvement:**
    - ______

- ○ ______

---

4. Web App 4-Axis Framework (Increase, Reduce, Introduce, Eliminate)

Objective:
Identify specific areas of the web app to amplify, simplify, add, or remove.

Key Discussion Points:

- **Increase:**
  - ______
  - ______
- **Reduce:**
  - ______
  - ______
- **Introduce:**
  - ______
  - ______
- **Eliminate:**
  - ______
  - ______

General Insights:

- ______
- ______

---

5. Prioritization & Impact Voting

Objective:
Determine which issues or features are most important by voting on the topics Increased by both groups.

Key Topics Voted On:

- **Top Priorities (Patients):**
  - ______
  - ______
- **Top Priorities (PTs):**
  - ______
  - ______

Voting Results:

- **Highest Voted Issues/Features:**
    - ______________________________
    - ______________________________
- **General Consensus:**
    - ______________________________
    - ______________________________

Final Thoughts/Comments:

- ______________________________
- ______________________________

---

6. Wrap Up

Objective:
Wrap up

Key Tasks Observed:

- Task:

Observations:

- Final Takeaway
    - ______________________________
    - ______________________________
- How we’re feeling today (record stakeholder one-word feels)
    - ______________________________
    - ______________________________

Summary and Next Steps:

Overall Impressions:

- ______________________________
- ______________________________

Recommendations for Follow-Up:

- ______________________________
- ______________________________
