# Supplementary material for: Co-designing meaningful extended reality for physical rehabilitation: a stakeholder-driven approach to embodied telehealth
Source: Front Virtual Real. Author manuscript; Available in PMC 2026 Aug 8. (PMC13450176; doi:10.3389/frvir.2025.1675309)
Supplement: Supplementary file 7 [file NIHMS2176626-supplement-Webapp-Clinician.pdf]

Web App Exploration Tasks - Clinician

The following recommended tasks are designed to help you explore and evaluate key features of the Immergo web app. Please complete the tasks below and circle their usability from 1 (Very Difficult) to 5 (Very Easy). Feel free to explore further into the prototypes as time allows.

Task:	Rating						
Create your avatar	(Very Difficult)	1	2	3	4	5	(Very Easy)
Find your patient	(Very Difficult)	1	2	3	4	5	(Very Easy)
Review patient measures	(Very Difficult)	1	2	3	4	5	(Very Easy)
Review appointment s	(Very Difficult)	1	2	3	4	5	(Very Easy)
Enable full body tracking	(Very Difficult)	1	2	3	4	5	(Very Easy)

Now ask your team coach to review early concepts through figma and discuss designs.

What tasks do you think are the most important beyond what's on this list? Share any additional notes below.
