# Supplementary material for: Co-designing meaningful extended reality for physical rehabilitation: a stakeholder-driven approach to embodied telehealth
Source: Front Virtual Real. Author manuscript; Available in PMC 2026 Aug 8. (PMC13450176; doi:10.3389/frvir.2025.1675309)

Webapp 4-Axis Evaluation

Raise
What should be emphasized or focused on because it works well or adds value?

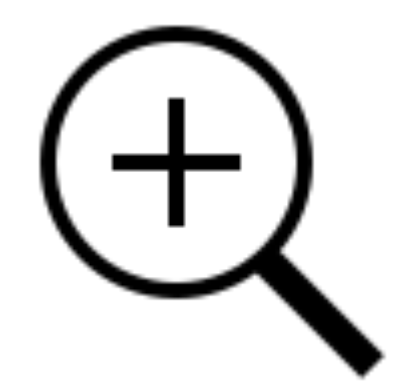

Reduce
What should be simplified or scaled back because its too complex or not as important?

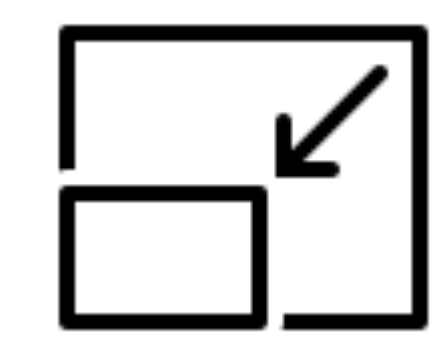

Create
What new features or tools should be added to improve the platform?

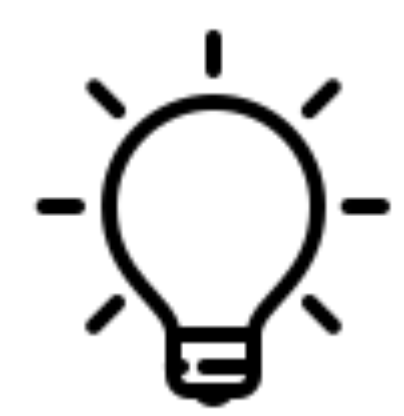

Eliminate
What should be removed because it doesn’t add value or is unnecessary?

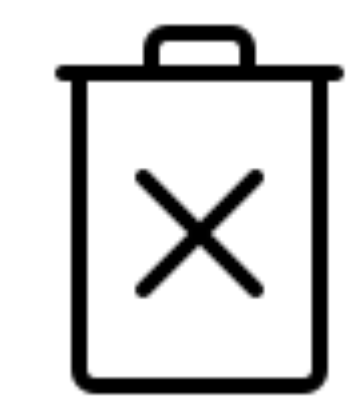

Supplement: Supplementary file 9 [file NIHMS2176626-supplement-Webapp_4_Axis_Framework.pdf]
